# Supplementary material for: Key stakeholders’ perspectives and experiences with defining, identifying and displaying gaps in health research: a qualitative study protocol
Source: BMJ Open. 2019 Sep 3;9(8):e027926. doi: 10.1136/bmjopen-2018-027926 (PMC6731867; doi:10.1136/bmjopen-2018-027926)
Supplement: Supplementary data [file bmjopen-2018-027926supp001.pdf]

Key stakeholders' perspectives and experiences with defining, identifying and displaying gaps in health research: a qualitative study protocol

Appendix 1: Semi-structured interview guide

Date: Interviewer: Archival #:

In person: Teleconference: Start Time: End Time:

Background?

1. Tell me a little about your work, and what you do?
   What does it involve?
2. Experience with using evidence for decision-making in health choices, policymaking, prioritizing research or funding projects?
3. How did you go about making the decision when the evidence was missing, insufficient or inadequate?

Defining research gaps

4. How would you describe the term "research gaps" in your own words?
   Probe based on participant (Researcher, Policy maker, Funder, Health Professional or Public/Patient)
   - *(Research) Can you walk me through how you use evidence to inform future research/research topics?*
   - *(Policy Makers) Can you walk me through how you use research to influence policies?*
   - *(Funders) Can you walk me through how you use research to determine which project to fund?*
   - *(Health Professionals) Can you walk me through how you use research to inform your practice as a health provider?*
   - *(Public/Patients) Can you walk me through how you use research to inform your health decisions?*
     - ✓ What are your thoughts on the importance of identifying research gaps?
     - ✓ What are your thoughts on the causes of research gaps?

Experiences, knowledge and perceived needs with methods used to identify research gaps

5. Could you talk about your views/any experience you have in identifying research gaps?
   - *(Research) For example, if you need to apply for funding, how would you select the study?*
   - *(Policy Makers) For example, if you work in developing policies?*
   - *(Funders) or example, if you need to fund projects, how do you determine which ones to fund?*
   - *(Health professionals) For example, in making decisions between treatment choices in your practice where there is uncertainty?*
   - *(Public/Patients) For example, when making health decisions where there is uncertainty?*

6. Could you tell me more specifically about the methods you used to identify research gaps?
   - ✓ What are some of the strengths of the method(s)/practices you used?
   - ✓ What are some of the challenges you experienced using the method(s) /practices?
7. Looking back on your experience using methods to identify research gaps, what is needed to improve the methods you used to identify research gaps?

Experiences, knowledge and perceived needs with methods used to display/present research gaps

8. Could you describe any experience you have in displaying/presenting research gaps?
9. Could you tell me more about the method(s) you used to display/present research gaps?
   - ✓ What are some of the strengths of the method(s) you used for displaying research gaps?
   - ✓ What are some of the challenges you experienced?
10. Please share any reflection on what you feel is needed to improve the methods you used to display/present research gaps?

General follow-up questions

11. Any additional thoughts you would like to share?

Nyanchoka L, *et al. BMJ Open* 2019; 9:e027926. doi: 10.1136/bmjopen-2018-027926

Key stakeholders' perspectives and experiences with defining, identifying and displaying gaps in health research: a qualitative study protocol

Appendix 2: Participant Information Sheet

Experiences with Methods for Identifying and Displaying Research Gaps

We invite you to take part in our research study. Before you decide whether to participate, you should understand why the research is being done and what it will involve. Please take your time to read the following information carefully and feel free to ask if you need more information or if there is anything that you do not understand. Please also feel free to discuss this with your friends, relatives and anyone else you wish.

What is the purpose of the study?

This study aims to explore the experiences of key stakeholders, including **the public, patients, researchers, clinicians, clinical guideline developers, public health professionals, policymakers and funders**, with methods for identifying and displaying research gaps, to inform **health choices, health practice, future research, policy or funding**. This study aims to help in better understanding the methods used to identify and display research gaps. The overall topic area on methods to identify and display gaps is still not well established, particularly because of no standard definition for the term "research gaps"; therefore a study to better understand the context, as well as the interactions of the factors such as alternative definitions, different audiences and methods used to identify gaps is important to improve our understanding of the audience's needs and the strengths and limitations of methods.

Why have I been chosen to take part?

You have been asked to take part because you are or have been involved in using research, producing research and/or communicating research. Your insight and experience with any methods you have used to identify and display research gaps will be highly appreciated to further guide this topic area.

Do I have to take part?

It is completely up to you whether or not you agree to take part in the study. If you do decide to take part, you will be asked to sign a consent form. If you decide to take part but then change your mind, you are free to do so at any time without giving a reason.

What will happen if I take part?

You will be asked to take part in an interview with a researcher, Linda Nyanchoka, about your experience with and your views of methods for identifying and display research gaps. The interviews will last approximately 20 to 40 minutes or as long as you would like to talk about your experience. With your permission, the interview will be audio-recorded. You can stop the interview at any time, and you do not have to answer a particular question if you don't want to.

Where will the interview take place?

The interview will take place in person at a specific location or over the phone. Participants in the UK have the option of an in-person or teleconference interview, and all other participants will have teleconference interviews at a date and time that is convenient for them.

Are there any risks in taking part?

We do not expect any risks or discomfort associated with this research study. However, if you feel uncomfortable, you can stop the interview at any time, without giving a reason.

Nyanchoka L, *et al. BMJ Open* 2019; 9:e027926. doi: 10.1136/bmjopen-2018-027926

Key stakeholders' perspectives and experiences with defining, identifying and displaying gaps in health research: a qualitative study protocol

Are there any benefits in taking part?

You will be helping develop our understanding of research gaps and methods for identifying and displaying research gaps.

Will my participation be kept confidential?

All the information you give us will be kept strictly confidential. The procedures for handling, processing, storing and destroying the data will comply with the Data Protection Act of 1998.

This means that only the researchers will see what you have said. The audio-recording of your interview will be identified by a code number only. These audio-recordings will be transcribed, and identifying details such as place names and people's names will be removed from the transcripts. We will use quotes from the interviews in the write-up of the study but will ensure that no one can be identified from these quotes.

At the end of the study, the research data, including consent forms, anonymised interview transcripts, field notes and your contact details, will be kept in locked filling cabinets and/or password-protected university computers for up to 10 years.

What will happen to the results of the study?

After the study has finished, the results will be written up as part of the PhD research thesis of Linda Nyanchoka and submitted for examination. The results will also be submitted for publication in an academic journal and presented at conferences.

If you would like to receive a copy of the findings, please let us know by using the contact information provided and we will happily provide you with one.

What will happen if I want to stop taking part?

If you decide at any point that you no longer wish to be part of the study, then you can withdraw without giving a reason. You can also ask for your data to be removed from the study and destroyed.

What if I am unhappy or if there is a problem?

If you are unhappy or if there is a problem, please feel free to let us know by contacting the lead researcher, Linda Nyanchoka, at the University of Liverpool (+33 75 34 29 417; L.Nyanchoka@liverpool.ac.uk). Linda will try to help or put you in touch with someone who can.

If you remain unhappy or have a complaint that you feel you cannot communicate to us, you should contact the Research Governance Officer at the University of Liverpool (0151 794 8290; ethics@liv.ac.uk). When contacting the Research Governance Officer, please provide the name or a description of the study (so that it can be identified), the researcher(s) involved, and the details of the complaint you wish to make.

Who is funding the research?

This research is funded by the European Union's Horizon 2020 research and innovation programme under the Marie Sklodowska-Curie grant (agreement no. 676207). If you want to find out more about the funding body, please contact https://ec.europa.eu/programmes/horizon2020/.

Who is doing this research?

The research and interviews will be conducted by Linda Nyanchoka, a Marie Curie Research Fellow at the University of Liverpool, UK.

Key stakeholders' perspectives and experiences with defining, identifying and displaying gaps in health research: a qualitative study protocol

How can I find out more?

You can get in touch with Linda Nyanchoka, who will be happy to answer any questions you might have:

Department of Biostatistics,

Institute of Translational Medicine

Block F/Waterhouse Buidling,

University of Liverpool,

Liverpool

L69 3BX

Teleconference no.: +33 75 34 29 417

Email address: lnyanchoka@gmail.com or
L.Nyanchoka@liverpool.ac.uk

Thank you for taking the time to read this document.

This information sheet is for you to keep

Nyanchoka L, *et al. BMJ Open* 2019; 9:e027926. doi: 10.1136/bmjopen-2018-027926

Key stakeholders' perspectives and experiences with defining, identifying and displaying gaps in health research: a qualitative study protocol

Appendix 3: Participant consent form

Researcher: Linda Nyanchoka

Please initial box

1. I confirm that I have read and have understood the information sheet dated [ ] for the above study. I have had the opportunity to consider the information, ask questions and have had these answered satisfactorily. ☐

2. I understand that my participation is voluntary and that I am free to withdraw at any time without giving any reason, without my rights being affected. ☐

3. I understand that, under the Data Protection Act 1998, I can at any time ask for access to the information I provide and I can also request the destruction of that information if I wish. ☐

4. I agree for the data I provide to be archived at The University of Liverpool. I understand that other authorised researchers will have access to this data only if they agree to preserve the confidentiality of the information as requested in this form. ☐

5. I agree to take part in the above study. ☐

________________________	_________	____________________
Participant name	Date	Signature
________________________	_________	____________________
Name of person taking consent	Date	Signature
________________________	_________	____________________
Researcher	Date	Signature

Nyanchoka L, *et al. BMJ Open* 2019; 9:e027926. doi: 10.1136/bmjopen-2018-027926

Key stakeholders' perspectives and experiences with defining, identifying and displaying gaps in health research: a qualitative study protocol

Principal Investigator

Catrin Tudur-Smith
University of Liverpool
Biostatistics Department
Block F Waterhouse Building
1-5 Brownlow
Liverpool L69 3GL

Tel: +44 (0)151 794 4059
Email: cat1@liverpool.ac.uk

Student Investigator

Linda Nyanchoka
University of Liverpool
Biostatistics Department
Block F Waterhouse Building
1-5 Brownlow Street
Liverpool L69 3GL

Tel: +33 75 34 29 417
Email: L.Nyanchoka@liverpool.ac.uk

The information you have submitted will be published as a report; please indicate whether you would like to receive a copy. ☐

I understand that confidentiality and anonymity will be maintained and it will not be possible to identify me in any publications ☐

I agree for the data collected from me to be used in future research and understand that any such use of identifiable data would be reviewed and approved by a research ethics committee. ☐

I understand and agree that my participation will be audio recorded and I am aware of and consent to your use of these recordings for the following purposes: meeting research aims and goals in exploring methods used to identify and display research gaps. ☐

I understand that the information collected about me will be used to support other research in the future, and may be shared anonymously with other researchers. ☐

I would like my name used and I understand and agree that what I have said or written as part of this study will be used in reports, publications and other research outputs so that anything I have contributed to this project can be recognised. ☐

I understand that my responses will be kept strictly confidential. I give permission for members of the research team to have access to my anonymised responses. I understand that my name will not be linked with the research materials, and I will not be identified or identifiable in the report or reports that result from the research. ☐

Nyanchoka L, *et al. BMJ Open* 2019; 9:e027926. doi: 10.1136/bmjopen-2018-027926

Key stakeholders' perspectives and experiences with defining, identifying and displaying gaps in health research: a qualitative study protocol

I understand and agree that once I submit my data it will become anonymised and I will therefore no longer be able to withdraw my data. ☐

I understand that the fully anonymised data will be held securely at the University of Liverpool and I can request access to the data collected, and/or request that the data is destroyed at any time until the data is submitted for publication. ☐

I understand that other authorised researchers may use my words in publications, reports, webpages, and other research outputs, only if they agree to preserve the confidentiality of the information as requested in this form. ☐

Nyanchoka L, *et al. BMJ Open* 2019; 9:e027926. doi: 10.1136/bmjopen-2018-027926

Key stakeholders' perspectives and experiences with defining, identifying and displaying gaps in health research: a qualitative study protocol

Appendix 4: Participant Teleconference Consent Form

Teleconference: Oral Consent Example Script:

Hello, I am Linda Nyanchoka, a PhD student from the University of Liverpool. I will be talking to you about my research project on defining research gaps and on methods to identify and display research gaps in health. Additional information is on the information sheet you have received.

Are you still interested in taking part in the project? *[Await confirmation]*. Now I'd like to confirm some of the details of the project to make sure you are clear about what's involved for you:

- We do not expect any risks or discomfort associated in this research study. However, if you feel uncomfortable, you can stop the interview at any time, without giving a reason.
- You do not have to say yes to take part; you can ask me any questions you want before or during the interview; you can also withdraw at any stage without giving a reason and without any negative consequences.
- You do not have to answer any questions that you do not wish to.
- You are aware that a University of Liverpool Research Ethics committee has approved this research project; for further information email me at L.Nyanchoka@liverpool.ac.uk
- I may use brief quotes of what you say during the interview in the write-up of this study, but they will remain anonymous.
- I will safely store your data electronically in encrypted, secure files. All identifiable data will be destroyed at the end of the study.
- I will audio-record you unless you say that I can't.
- Are you still willing to take part?
  Do you give your permission for me to re-contact you to clarify information?

[*Await confirmation]* So if you're happy with all of that, and have no more questions, let's start.

Researcher: Linda Nyanchoka

Participant:

Date:

Time:

Nyanchoka L, *et al. BMJ Open* 2019; 9:e027926. doi: 10.1136/bmjopen-2018-027926
