# Supplementary material for: Key stakeholders’ perspectives and experiences with defining, identifying and displaying gaps in health research: a qualitative study protocol
Source: BMJ Open. 2019 Sep 3;9(8):e027926. doi: 10.1136/bmjopen-2018-027926 (PMC6731867; doi:10.1136/bmjopen-2018-027926)
Supplement: Author's manuscript [file bmjopen-2018-027926.draft_revisions.pdf]

BMJ Open is committed to open peer review. As part of this commitment we make the peer review history of every article we publish publicly available.

When an article is published we post the peer reviewers' comments and the authors' responses online. We also post the versions of the paper that were used during peer review. These are the versions that the peer review comments apply to.

The versions of the paper that follow are the versions that were submitted during the peer review process. They are not the versions of record or the final published versions. They should not be cited or distributed as the published version of this manuscript.

BMJ Open is an open access journal and the full, final, typeset and author-corrected version of record of the manuscript is available on our site with no access controls, subscription charges or pay-per-view fees (http://bmjopen.bmj.com).

If you have any questions on BMJ Open's open peer review process please email
info.bmjopen@bmj.com

Exploring Key Stakeholder Experiences with Defining, Identifying and Displaying Gaps in Health Research: A Qualitative Study Protocol

Journal:	*BMJ Open*
Manuscript ID	bmjopen-2018-027926
Article Type:	Protocol
Date Submitted by the Author:	15-Nov-2018
Complete List of Authors:	Nyanchoka, Linda; Université Paris Descartes, ; University of Liverpool, Institute of Translational Medicine<br>Tudur-Smith, Catrin; University of Liverpool, Biostatistics<br>Porcher, Raphaël; Inserm U1153<br>Hren, Darko; University of Split, School of Humanities and Social Sciences
Keywords:	Evidence Synthesis; Knowledge Synthesis; Scoping Review; Evidence Mapping; Gaps in Clinical Research; Treatment Uncertainties, QUALITATIVE RESEARCH, Research Uncertainties, Research Gaps; Research Priorities; Displaying Gaps; Evidence-based Decision-making; Evidence-based Care, Evidence-based Research

Title

Exploring Key Stakeholder Experiences with Defining, Identifying and Displaying Gaps in Health Research: A Qualitative Study Protocol

Authors

Linda Nyanchoka[1, 2, 3], Catrin Tudur-Smith[3], Raphaël Porcher[1, 2, 4], Darko Hren[5]

Affiliations

1. Université Paris Descartes, Sorbonne Paris Cité, Faculté de Médecine, Paris, France
2. INSERM, UMR1153, Epidemiology and Statistics Sorbonne Paris Cité Research Center (CRESS), Team METHODS, Paris, France
3. University of Liverpool, Institute of Translational Medicine, Liverpool, United Kingdom
4. Assistance Publique des Hôpitaux de Paris (AP-HP), Hôpital Hôtel-Dieu, Center for Clinical Epidemiology, Paris, France
5. University of Split, Department of Psychology, Faculty of Humanities and Social Sciences, Split, Croatia

Keywords

Evidence Synthesis; Knowledge Synthesis; Scoping Review; Evidence Mapping; Gaps in Clinical Research; Treatment Uncertainties; Research Gaps; Research Priorities; Displaying Gaps; Evidence-based Decision-making; Evidence-based Care; Evidence-based Research

Correspondence

Linda Nyanchoka lnyanchoka@gmail.com

1. Université Paris Descartes, Sorbonne Paris Cité, Faculté de Médecine, Paris, France
2. INSERM, UMR1153, Epidemiology and Statistics Sorbonne Paris Cité Research Center (CRESS), Team METHODS, Paris, France
3. University of Liverpool, Institute of Translational Medicine, Liverpool, United Kingdom

Word Count: 2247

For peer review only

Abstract

Introduction

Identifying research gaps can inform the design and conduct of health research, practice and policies. Audiences including the public, patients, researchers, clinical guideline developers, clinicians, policymakers, research regulatory bodies and funders can also benefit from understanding the status of research and research gaps to make informed choices. This study aims to explore key informants' knowledge and experiences with defining research gaps and approaches for identifying and displaying research gaps in health research, practice and policy.

Methods and analysis

This is an exploratory qualitative study using semi-structured in-depth interviews. The participants will be recruited by use of convenience and snowball sampling from initiatives and organizations related to health research, practice and policies. We anticipate performing approximately 14 to 28 interviews from the different key informant groups (the public, patients, researchers, clinicians, clinical guideline developers, public health professionals, policymakers and funders). Interviews will be thematically analysed following the method outlined by Braun and Clarke. The qualitative data-analysis software NVivo 12 Pro will be used to aid data management and analysis.

Ethics and dissemination

The research has obtained ethical approval from the University of Liverpool, UK. The findings will be disseminated via conferences, workshops, meetings and peer-reviewed publications.

For peer review only

For peer review only - http://bmjopen.bmj.com/site/about/guidelines.xhtml

Strengths and Limitations

- This study gathers views on defining research gaps and approaches to identifying and displaying research gaps in health.
- Qualitative interview data will be thematically analysed to form the basis of the key stakeholder experiences related to defining, identifying and displaying research gaps in health research, practice and policy.
- This study is a follow-up study to a scoping review that described the methods used to identify and display research gaps reported in scientific publications.
- The study is limited to research gaps in the field of health.

For peer review only

For peer review only - http://bmjopen.bmj.com/site/about/guidelines.xhtml

1. Background

The term "research gap" is not well defined and its meaning can differ depending on the research context. In this study, we adopted the definition from the National Collaborating Centre for Methods and Tools (NCCMT) in Canada, which describes a research gap as a clinical question for which missing or insufficient information limits the ability to reach a conclusion [1].

Investigating suitable approaches to evaluate the current state of scientific knowledge would help the public, health professionals, researchers and decision-makers understand areas of uncertainty within the research problem and topic area [2]. Healthcare decisions for individual patients, public health policies, and clinical guidelines should be informed by the best available research evidence while taking into consideration missing, inadequate and insufficient evidence. Identifying research gaps enables researchers to ascertain the research problem and scope of the study, which in turn is the key to success in a research project, informed by input from previous research studies. It also allows patients, the public, clinicians and decision-makers to make informed decisions by knowing the areas of uncertainty in research.

Systematic reviews have been considered the standard methods for identifying research gaps [1]. In this study we aim to explore other ways and methods that are being used to define, identify and display gaps in health research. This exploration will improve the understanding of how different approaches influence research planning, health practice, and policy. To the best of our knowledge, this is the first study that aims to explore experiences with describing, identifying and displaying health research gaps from the perspective of different key stakeholders including the public, patients, researchers, clinicians, clinical guideline developers, public health professionals, policymakers, and funders.

The specific objectives of the study are to 1) investigate key stakeholders' views on describing health research gaps and 2) explore key stakeholders' experiences with identifying and displaying health research gaps to make informed decisions and inform further research, practice and policy.

For peer review only - http://bmjopen.bmj.com/site/about/guidelines.xhtml

For peer review only

2. Study Design

This study is an exploratory qualitative study using semi-structured in-depth interviews. This design is considered the most suitable because the qualitative approach facilitates an exploration of a phenomenon within its context. Moreover, the qualitative approach allows a researcher to investigate phenomena in which all the relevant information or issues cannot be anticipated (e.g., context-specific, little-known, or newly emerging phenomena). Qualitative methods are used to identify variations in ideas and bring out details of the context and events, reasons for practices, and barriers and difficulties in a specific context. They can also be used to identify the possible relationships between factors and issues [2]. Investigating perspectives of different stakeholders will ensure that the issue is not explored through one lens but rather a variety of lenses, which allows for multiple facets of the phenomenon to be revealed and understood [3]. In addition, the qualitative approach provides more in-depth and comprehensive information accompanied by a wide understanding of the entire situation [3].

2.1 Interviews (in-person and teleconference)

The semi-structured interviews will focus on the public, patients, researchers, clinicians, clinical guideline developers, public health professionals, policymakers and funders. Semi-structured interviews are an appropriate method in this instance to gather a deeper understanding of an unclear topic area. The use of semi-structured interviews allows for specific areas to be addressed while giving the interviewees the opportunity to talk about unforeseen areas that are important to them and may not have been explored or anticipated by the researcher(s) [4].

This study will involve both teleconference and in-person interviews. In-person interviews will be conducted primarily with participants residing or reachable in London, UK, and global participants will be interviewed via teleconference (see Appendix 1 for the interview guide for both the in-person and teleconference interviews). The topic guide may be further developed or adjusted after initial interviews on the basis of participant responses that can be useful to gather more comprehensive

For peer review only - http://bmjopen.bmj.com/site/about/guidelines.xhtml

information. The guide was developed by focusing on exploring key stakeholder experiences with the following key areas:

1) Participant background information
2) Definitions of research gaps
3) Experience with identifying and displaying research gaps to inform further research, practice and policy

2.2 Key Informants

The key informants for our study were selected on the basis of their involvement in activities in which research is used to inform further health research, practice and policies. The participants will be recruited by using snowballing and convenience sampling, mainly from initiatives and organizations related to health research, practice and policies. More information and examples of organizations are given in Table 1. Participants will be considered eligible if they have used, developed, disseminated or commissioned health research. We anticipate performing about 14 to 28 interviews. The selection and number of interviews chosen aims to obtain a varied scope of responses from each category with a goal of attaining data saturation (i.e., the point when new data do not add to a better understanding of studied phenomenon but rather repeat what was previously expressed [5]). Saturation will be guided by the seven parameters identified by Hennink et al., [6] including the study purpose, population, sampling strategy, data quality, type of codes, code book and saturation goal and focus retrieved from the study. These parameters will be taken into consideration throughout the study.

Table 1. Key Informant

Categories	Key Informants	Examples of Organizations	Expected Number of Interviews
Health research	Researchers	- Research institutes/universities (lead researchers)<br>- Cochrane Priority Setting Methods Group<br>- Knowledge synthesis research groups	2-4
	Funding bodies	- UK National Institute for Health Research<br>- European Union	2-4

For peer review only - http://bmjopen.bmj.com/site/about/guidelines.xhtml

Health practice	Public, patients	- James Lind Alliance	2-4
	Clinicians	- UK National Health Service	2-4
	Clinical guideline developers	UK National Institute for Health and Care Excellence	2-4
	Public health professionals	- National public health bodies (e.g., Public Health England)	2-4
Policymaking	Policymakers	- UK National Health Service<br>- Ministry of Health officials	2-4

3. Data Analysis

3.1 Transcription

All interviews will be transcribed verbatim and anonymised. The lead researcher (LN) will transcribe two interviews to help inform the analytic process, and the other audio files will be transcribed by a professional transcription agency licensed from the University of Liverpool.

3.2 Thematic Data Analysis

Interviews will be thematically analysed in accordance with the steps outlined by Braun and Clarke [7]. The steps include the following: 1) open coding from interview responses will be performed by two researchers independently; 2) corroboration of initial codes will be discussed among the research team and an initial codebook will be developed; 3) the code structure will be used for analysing the remaining responses with openness to including new codes and refining existing ones; and 4) themes and subthemes will be identified from the final code structure and their relationships will be represented by using a thematic map [7]. Trustworthiness during thematic data analysis will be ensured by storing raw data in well-organized archives, documenting detailed notes about the development and hierarchies of concepts and themes, establishing consensus on themes, and providing detailed descriptions of context, and describing the process of coding and analysis with sufficient detail [8, 9]. NVivo 12 Pro, a qualitative data analysis software, will be used for data management and analysis.

For peer review only

For peer review only - http://bmjopen.bmj.com/site/about/guidelines.xhtml

The interviews will be recorded on a digital recorder for face-to-face interviews and electronically for teleconference interviews. The recorded data will be transcribed. We will use analytical categories to describe and explain experiences reported among the different groups of participants. The categories will be inductively derived from the data gathered by the semi-structured interviews. All data relevant to each category will be identified and examined thoroughly to develop relevant themes. This examination requires a coherent and systematic approach and involves adding categories to reflect as many of the nuances in the data as possible, rather than reducing the data. All data relevant to each category (describing research gaps, experience with identifying and displaying research gaps) will be identified and examined to ensure that each data item is checked accordingly.

4. Ensuring study quality

To further ensure rigour and trustworthiness, this study will be guided by Guba and Lincoln's concepts for defining and investigating quality in qualitative research that can be considered parallel to quantitative research concepts of validity and reliability [6, 8, 10]. The concepts include credibility, transferability, dependability, confirmability, audit trails and reflectivity.

According to Tobin and Begley, credibility is the interaction between respondents' views and the researcher's perspectives of them [11]. One of the ways we aim to ensure credibility is by planning debriefing sessions between the lead researcher (LN) and senior researcher (DH) to provide a sounding board for LN on elaborating different study ideas and interpretations [6]. Transferability refers to the generalizability of the study findings [3, 8]. We aim to address this criterion by reporting the rich background and context descriptions of our study findings from the different key stakeholders. Dependability involves participants' evaluation of the findings, interpretation and recommendations of the study [8]. To take this into consideration, we aim to clearly outline the different steps of the project and its findings. Confirmability refers to establishing that data collection and interpretations of the study are clearly deliberated from the data and not misinterpreted [8]. We will ensure confirmability by documenting the justification of methodological and analytical choices to illustrate how the data were derived in relation to the study objectives. Audit trails refer to

For peer review only - http://bmjopen.bmj.com/site/about/guidelines.xhtml

transparently describing the research steps taken from the start of the project to the development and reporting of the findings [8]. Records of the research path will be kept throughout the study. Finally, reflexivity includes examining one's own conceptual lens, explicit and implicit assumptions, preconceptions and values and how these affect research decisions in all phases of qualitative studies [8]. Reflexivity will be assured by maintaining a reflexive journal to document the different thoughts and reflections about the study process. These concepts are interrelated, and thinking through them from the onset and incorporating them in a study improves the study rigor.

5. Patient and Public Involvement

This study will include patients and the members of the public as key informants interviewed. Such participants will allow us to better understand their perceived needs and priorities in identifying research gaps to make informed health decisions. We will also be able to compare and cross-check the public's and patients' views and experiences in relation to the other key informants.

6. Expected Outcomes

The study will provide insights into issues related to defining research gaps and methods used to identify and display gaps in health research from perspectives of key stakeholders involved in the process. This is a follow-up study of a wider project; the first study was a scoping review exploring methods used to identify and display research gaps as reported in scientific publications. This is the protocol for the second study that aims to complement and enrich the findings of the scoping review by investigating key stakeholder experiences with identifying and displaying research gaps. The aim of the final step in the overall project will be to develop methodological guidance on methods for identifying and displaying gaps in health research.

7. Ethics

7.1 Ethical approval

The research has obtained ethical approval from the University of Liverpool, UK.

For peer review only - http://bmjopen.bmj.com/site/about/guidelines.xhtml

7.2 Informed consent

Informed consent will be carried out in accordance with the University of Liverpool Ethics Committee board requirements. Verbal consent will be sought for phone interviews and written consent for in-person interviews.

7.3 Confidentiality and data protection

Confidentiality and data protection will be undertaken in accordance with the University of Liverpool, Ethics Committee board. All participant information will be anonymized, and hard-copy data will be stored in a locked unit. Soft-copy material will be stored in a password-protected file. Upon completion of the study and publication of the study results, all study material will be stored and disposed of according to the rules and regulations of the University of Liverpool. The study protocol will be stored on the data repository Zenodo.

8. Dissemination

This study will help better understand how different stakeholders define research gaps and the methods they use to identify and display research gaps. The overall topic area of methods to identify and display gaps is still not well established, particularly because of no standard definition for the term "research gaps". Therefore, a study to better understand the context and interactions of factors such as alternative definitions, different audiences and methods used to identify gaps is important to improve our understanding of the key stakeholders' experiences with different methods. At the end of this research project, the results will be presented at conferences and relevant meetings. They will also be written up for publication in a peer-reviewed journal and as part of a doctoral thesis of the PhD fellow (LN).

9. Funding

This project is a part of a MiRoR (Methods in Research on Research)-funded PhD being undertaken by LN. MiRoR received funding from the European Union's Horizon 2020 research and innovation programme under a Marie Sklodowska-Curie grant (agreement no. 676207).

For peer review only - http://bmjopen.bmj.com/site/about/guidelines.xhtml

10. Conflict of interest

The authors declare no conflicts of interest.

11. Authors' contributions

LN conceived the study with guidance and feedback from DH, RP and CTM. All authors read and approved the final manuscript.

12. Acknowledgements

We acknowledge the feed-back and support of Daniela Lai and Cristian R. Montenegro in critiquing the interview guide.

For peer review only

References

1. Robinson KA, Saldanha IJ, Mckoy NA: **Frameworks for determining research gaps during systematic reviews**. 2011.

2. Bouma GD, Ling R: **The research process**: Oxford University Press, USA; 2004.

3. Yin RK: **Case study research: Design and methods (applied social research methods)**. *London and Singapore: Sage* 2009.

4. Britten N: **Qualitative research: qualitative interviews in medical research**. *Bmj* 1995, **311**(6999):251-253.

5. Saunders B, Sim J, Kingstone T, Baker S, Waterfield J, Bartlam B, Burroughs H, Jinks C: **Saturation in qualitative research: exploring its conceptualization and operationalization**. *Quality & Quantity* 2018, **52**(4):1893-1907.

6. Shenton AK: **Strategies for ensuring trustworthiness in qualitative research projects**. *Education for information* 2004, **22**(2):63-75.

7. Braun V, Clarke V: **Using thematic analysis in psychology**. *Qualitative research in psychology* 2006, **3**(2):77-101.

8. Inquiry N: **Newbury Park**. *Calif: Sage Publications* 1985.

9. Nowell LS, Norris JM, White DE, Moules NJ: **Thematic analysis: Striving to meet the trustworthiness criteria**. *International Journal of Qualitative Methods* 2017, **16**(1):1609406917733847.

10. Glonti K, Hren D: **Editors' perspectives on the peer-review process in biomedical journals: protocol for a qualitative study**. *BMJ Open* 2018, **8**(10).

11. Tobin GA, Begley CM: **Methodological rigour within a qualitative framework**. *Journal of advanced nursing* 2004, **48**(4):388-396.

For peer review only - http://bmjopen.bmj.com/site/about/guidelines.xhtml

For peer review only

Appendix 1. Semi-structured interview guide

Experience with Methods for Identifying and Displaying Research Gaps in Health Research

Date: Interviewer:

Archival #: In person: Teleconference: Start Time: End Time:

Background (information about participant's experience in using evidence)

1. Tell me a little about your work, and what you do?

What does it involve?

2. Experience with using evidence for decision-making in health choices, policymaking, prioritizing research or funding projects?

3. How did you go about making the decision when the evidence was missing, insufficient or inadequate?

Explanations of research gaps

4. In your line of work and experience, how would you describe the term "research gaps"?
   - ✓ *What are your thoughts on the importance of identifying research gaps?*
   - ✓ *What are your thoughts on the causes of research gaps?*

Experience with methods to identify research gaps

5. Could you talk about any experience you have in identifying research gaps?

6. Could you tell me more specifically about the methods you used to identify research gaps?
   - ✓ *What are some of the challenges you experienced?*
   - ✓ *What are some of the strengths of the method(s) you used for identifying research gaps?*

7. Looking back on your experience using methods to identify research gaps, what is needed to improve the methods you used to identify research gaps?

Experience with methods to display/present research gaps

8. Could you describe any experience you have in displaying/presenting research gaps?

9. Could you tell me more about the method(s) you used to display/present research gaps?
   - ✓ *What are some of the strengths of the method(s) you used for displaying research gaps?*
   - ✓ *What are some of the challenges you experienced?*

10. Please share any reflection on what you feel is needed to improve the methods you used to display/present research gaps?

General follow-up questions

11. Any additional thoughts you would like to share concerning research gaps or methods to identify and display research gaps?

For peer review only

Appendix 2. Participant Information Sheet

Experiences with Methods for Identifying and Displaying Research Gaps

We invite you to take part in our research study. Before you decide whether to participate, you should understand why the research is being done and what it will involve. Please take your time to read the following information carefully and feel free to ask if you need more information or if there is anything that you do not understand. Please also feel free to discuss this with your friends, relatives and anyone else you wish.

What is the purpose of the study?

This study aims to explore the different experiences of key stakeholders, including **the public, patients, researchers, clinicians, clinical guideline developers, public health professionals, policymakers and funders**, with methods for identifying and displaying research gaps, to inform **health choices, health practice, future research, policy or funding**. This study aims to help in better understanding the different methods used to identify and display research gaps. The overall topic area on methods to identify and display gaps is still not well established, particularly because of no standard definition for the term "research gaps"; therefore a study to better understand the context, as well as the interactions of the factors such as alternative definitions, different audiences and methods used to identify gaps is important to improve our understanding of the audience's needs and the strengths and limitations of different methods.

Why have I been chosen to take part?

You have been asked to take part because you are or have been involved in using research, producing research and/or communicating research. Your insight and experience with any methods you have used to identify and display research gaps will be highly appreciated to further guide this topic area.

Do I have to take part?

It is completely up to you whether or not you agree to take part in the study. If you do decide to take part, you will be asked to sign a consent form. If you decide to take part but then change your mind, you are free to do so at any time without giving a reason.

What will happen if I take part?

You will be asked to take part in an interview with a researcher, Linda Nyanchoka, about your experience with and your views of methods for identifying and display research gaps. The interviews will last approximately 20 to 40 minutes or as long as you would like to talk about your experience. With your permission, the interview will be audio-recorded. You can stop the interview at any time, and you do not have to answer a particular question if you don't want to.

Where will the interview take place?

The interview will take place in person at a specific location or over the phone. Participants in the UK have the option of an in-person or teleconference interview, and all other participants will have teleconference interviews at a date and time that is convenient for them.

Are there any risks in taking part?

We do not expect any risks or discomfort associated with this research study. However, if you feel uncomfortable, you can stop the interview at any time, without giving a reason.

For peer review only

Are there any benefits in taking part?

You will be helping develop our understanding of research gaps and methods for identifying and displaying research gaps.

Will my participation be kept confidential?

All the information you give us will be kept strictly confidential. The procedures for handling, processing, storing and destroying the data will comply with the Data Protection Act of 1998.

This means that only the researchers will see what you have said. The audio-recording of your interview will be identified by a code number only. These audio-recordings will be transcribed, and identifying details such as place names and people's names will be removed from the transcripts. We will use quotes from the interviews in the write-up of the study but will ensure that no one can be identified from these quotes.

At the end of the study, the research data, including consent forms, anonymised interview transcripts, field notes and your contact details, will be kept in locked filling cabinets and/or password-protected university computers for up to 10 years.

What will happen to the results of the study?

After the study has finished, the results will be written up as part of the PhD research thesis of Linda Nyanchoka and submitted for examination. The results will also be submitted for publication in an academic journal and presented at conferences.

If you would like to receive a copy of the findings, please let us know by using the contact information provided and we will happily provide you with one.

What will happen if I want to stop taking part?

If you decide at any point that you no longer wish to be part of the study, then you can withdraw without giving a reason. You can also ask for your data to be removed from the study and destroyed.

What if I am unhappy or if there is a problem?

If you are unhappy or if there is a problem, please feel free to let us know by contacting the lead researcher, Linda Nyanchoka, at the University of Liverpool (+33 75 34 29 417; L.Nyanchoka@liverpool.ac.uk). Linda will try to help or put you in touch with someone who can.

If you remain unhappy or have a complaint that you feel you cannot communicate to us, you should contact the Research Governance Officer at the University of Liverpool (0151 794 8290; ethics@liv.ac.uk). When contacting the Research Governance Officer, please provide the name or a description of the study (so that it can be identified), the researcher(s) involved, and the details of the complaint you wish to make.

Who is funding the research?

This research is funded by the European Union's Horizon 2020 research and innovation programme under the Marie Sklodowska-Curie grant (agreement no. 676207). If you want to find out more about the funding body, please contact https://ec.europa.eu/programmes/horizon2020/.

Who is doing this research?

The research and interviews will be conducted by Linda Nyanchoka, a Marie Curie Research Fellow at the University of Liverpool, UK.

For peer review only

How can I find out more?

You can get in touch with Linda Nyanchoka, who will be happy to answer any questions you might have:

Department of Biostatistics,

Institute of Translational Medicine

Block F/Waterhouse Buidling,

University of Liverpool,

Liverpool

L69 3BX

Teleconference no.: +33 75 34 29 417

Email address: lnyanchoka@gmail.com or L.Nyanchoka@liverpool.ac.uk

Thank you for taking the time to read this document.

This information sheet is for you to keep

For peer review only

Appendix 3. Participant consent form

Title of the research project: **Experiences on Methods for Identifying and Displaying Research Gaps**

Researcher: Linda Nyanchoka

Please initial box

1. I confirm that I have read and have understood the information sheet dated [ ] for the above study. I have had the opportunity to consider the information, ask questions and have had these answered satisfactorily. ☐
2. I understand that my participation is voluntary and that I am free to withdraw at any time without giving any reason, without my rights being affected. In addition, should I not wish to answer any particular question or questions, I am free to decline. ☐
3. I understand that, under the Data Protection Act 1998, I can at any time ask for access to the information I provide and I can also request the destruction of that information if I wish. ☐
4. I agree for the data I provide to be archived at The University of Liverpool. I understand that other authorised researchers will have access to this data only if they agree to preserve the confidentiality of the information as requested in this form. ☐
5. I agree to take part in the above study. ☐

______________________	________	______________________
Participant name	Date	Signature
______________________	________	______________________
Name of person taking consent	Date	Signature
______________________	________	______________________
Researcher	Date	Signature

For peer review only

Principal Investigator

Catrin Tudur-Smith
University of Liverpool
Biostatistics Department
Block F Waterhouse Building
1-5 Brownlow
Liverpool L69 3GL

Tel: +44 (0)151 794 4059
Email: cat1@liverpool.ac.uk

Student Investigator

Linda Nyanchoka
University of Liverpool
Biostatistics Department
Block F Waterhouse Building
1-5 Brownlow Street
Liverpool L69 3GL

Tel: +33 75 34 29 417
Email: L.Nyanchoka@liverpool.ac.uk

The information you have submitted will be published as a report; please indicate whether you would like to receive a copy. ☐

I understand that confidentiality and anonymity will be maintained and it will not be possible to identify me in any publications ☐

I agree for the data collected from me to be used in future research and understand that any such use of identifiable data would be reviewed and approved by a research ethics committee. ☐

I understand and agree that my participation will be audio recorded and I am aware of and consent to your use of these recordings for the following purposes: meeting research aims and goals in exploring methods used to identify and display research gaps. ☐

I understand that the information collected about me will be used to support other research in the future, and may be shared anonymously with other researchers. ☐

I would like my name used and I understand and agree that what I have said or written as part of this study will be used in reports, publications and other research outputs so that anything I have contributed to this project can be recognised. ☐

I understand that my responses will be kept strictly confidential. I give permission for members of the research team to have access to my anonymised responses. I understand that my name will not be linked with the research materials, and I will not be identified or identifiable in the report or reports that result from the research. ☐

For peer review only

I understand and agree that once I submit my data it will become anonymised and I will therefore no longer be able to withdraw my data. ☐

I understand that the fully anonymised data will be held securely at the University of Liverpool and I can request access to the data collected, and/or request that the data is destroyed at any time until the data is submitted for publication. ☐

I understand that other authorised researchers may use my words in publications, reports, webpages, and other research outputs, only if they agree to preserve the confidentiality of the information as requested in this form. ☐

For peer review only

Appendix 4. Participant Teleconference Consent Form

Teleconference: Oral Consent Example Script:

Hello, I am Linda Nyanchoka, a PhD student from the University of Liverpool. I will be talking to you about my research project on defining research gaps and on methods to identify and display research gaps in health. Additional information is on the information sheet you have received.

Are you still interested in taking part in the project? *[Await confirmation]*. Now I'd like to confirm some of the details of the project to make sure you are clear about what's involved for you:

- We do not expect any risks or discomfort associated in this research study. However, if you feel uncomfortable, you can stop the interview at any time, without giving a reason.
- You do not have to say yes to take part; you can ask me any questions you want before or during the interview; you can also withdraw at any stage without giving a reason and without any negative consequences.
- You do not have to answer any questions that you do not wish to.
- You are aware that a University of Liverpool Research Ethics committee has approved this research project; for further information email me at L.Nyanchoka@liverpool.ac.uk
- I may use brief quotes of what you say during the interview in the write-up of this study, but they will remain anonymous.
- I will safely store your data electronically in encrypted, secure files. All identifiable data will be destroyed at the end of the study.
- I will audio-record you unless you say that I can't.
- Are you still willing to take part?
  Do you give your permission for me to re-contact you to clarify information?

[*Await confirmation]* So if you're happy with all of that, and have no more questions, let's start.

Researcher: Linda Nyanchoka

Participant:

Date:

Time:

For peer review only

BMJ Open

Key stakeholders' perspectives and experiences with defining, identifying and displaying gaps in health research: a qualitative study protocol

Journal:	*BMJ Open*
Manuscript ID	bmjopen-2018-027926.R1
Article Type:	Protocol
Date Submitted by the Author:	10-Apr-2019
Complete List of Authors:	Nyanchoka, Linda; Université Paris Descartes, ; University of Liverpool, Institute of Translational Medicine<br>Tudur-Smith, Catrin; University of Liverpool, Biostatistics<br>Porcher, Raphaël; Inserm U1153<br>Hren, Darko; University of Split, School of Humanities and Social Sciences
**Primary Subject Heading**:	Qualitative research
Secondary Subject Heading:	Research methods
Keywords:	Research Uncertainties, Identifying Research Gaps, Displaying Research Gaps, Evidence Synthesis, Evidence-based Decision-making, QUALITATIVE RESEARCH

SCHOLARONE™
Manuscripts

Title

Key stakeholders' perspectives and experiences with defining, identifying and displaying gaps in health research: a qualitative study protocol

Authors

Linda Nyanchoka[1, 2, 3], Catrin Tudur-Smith[3], Raphaël Porcher[1, 2, 4], Darko Hren[5]

Affiliations

1. Université Paris Descartes, Sorbonne Paris Cité, Faculté de Médecine, Paris, France
2. INSERM, UMR1153, Epidemiology and Statistics Sorbonne Paris Cité Research Center (CRESS), Team METHODS, Paris, France
3. University of Liverpool, Institute of Translational Medicine, Liverpool, United Kingdom
4. Assistance Publique des Hôpitaux de Paris (AP-HP), Hôpital Hôtel-Dieu, Center for Clinical Epidemiology, Paris, France
5. University of Split, Department of Psychology, Faculty of Humanities and Social Sciences, Split, Croatia

Keywords

Evidence Synthesis; Research Gaps; Research Priorities; Identifying Research gaps; Displaying Research Gaps; Evidence-based decision-making; Qualitative Study

Correspondence

Linda Nyanchoka lnyanchoka@gmail.com

1. Université Paris Descartes, Sorbonne Paris Cité, Faculté de Médecine, Paris, France
2. INSERM, UMR1153, Epidemiology and Statistics Sorbonne Paris Cité Research Center (CRESS), Team METHODS, Paris, France
3. University of Liverpool, Institute of Translational Medicine, Liverpool, United Kingdom

Word Count: Abstract: 282 Manuscript: 4683

For peer review only

Abstract

Introduction

Identifying research gaps can inform the design and conduct of health research, practice and policies by informing the current body of evidence. Audiences including the researchers, clinical guideline developers, clinicians, policymakers, research regulatory bodies, funders and public/patients can also benefit from understanding the status of research and research gaps to make informed choices. This study aims to explore how key informants define research gaps and characterize methods/practices used to identify and display gaps in health research to inform future research practice and policies.

Methods and analysis

This is an exploratory qualitative study using semi-structured in-depth interviews. The participants will be recruited by purposive sampling from initiatives and organizations previously identified in a scoping review on methods to identify, prioritize and display gaps in health research. We anticipate performing up to 28 interviews with the different key informant groups involved in using evidence to inform health practice, policy making and research planning (i.e. researchers, clinicians, clinical guideline developers, public health professionals, policymakers, public/patients, and funders). Interviews will be thematically analysed as outlined by Braun and Clarke. The qualitative data-analysis software NVivo 12 Pro will be used to aid data management and analysis.

Discussion

This is the protocol for a follow-up study that aims to complement and enrich the findings of the scoping review on methods to identify, prioritize and display gaps in health research. The overall project aims to develop methodological guidance for identifying gaps in health research.

Ethics and dissemination

The research obtained ethical approval from the University of Liverpool, UK. The findings will be disseminated via conferences, meetings (organized by the Methods in Research on Research project),

For peer review only - http://bmjopen.bmj.com/site/about/guidelines.xhtml

peer-reviewed publications and lay magazines because the study participants will include public/patients.

Strengths and limitations

- This study will explore how key informants define research gaps and what formal and informal practices they use to identify and display gaps in health research, practice, and policy.
- This study will contribute to the development of methodological guidance to describe, identify and display gaps in health research.
- This study is informed by and complements a previously conducted scoping review on methods to identify, prioritize and display gaps in health research.
- This would have benefited from including patients/public perspectives in designing the study to be able to improve the importance and relevance of the findings for this population.

eview only

For peer review only - http://bmjopen.bmj.com/site/about/guidelines.xhtml

BACKGROUND

Identifying research gaps can help inform the design and conduct of health research, practice and policies by providing a better understanding of the current body of evidence. The term "research gap" is not well defined, and its meaning can differ depending on the researcher and research context. A recent scoping review on methods used to identify, prioritize and display gaps in health research reported 12 different definitions related to gaps in health research (e.g., population, theoretical and methodology gaps), each describing research gaps differently [1]. This finding shows the ambiguity of the term "research gaps" and the different practices it may be related to.

As a basis for further exploring and understanding "research gaps", we start from the definition given by the National Collaborating Center for Methods and Tools (NCCMT) in Canada based on the work of Robinson et al., whereby a research gap is defined as a topic or area for which missing or insufficient information limits the ability to reach a conclusion for a question [2]. Given the different meanings and definitions of research gaps found in the scoping review [1], we consider it important to further explore definitions rather than just adopt or modify the NCCMT definition. Clearly defining the type of research gap can help determine how to better identify, characterize, prioritize and address research gaps.

A study on "Identifying and Prioritizing Research Gaps" corroborated that systematic reviews are the standard for evaluating the existing state of scientific knowledge regarding a specific clinical or policy question [3]. Robinson et al., also developed a framework using systematic reviews to identify research gaps [2] in which they classified the reasons for existence of research gaps and the use of Population, Intervention, Comparison, Outcome and Setting (PICOS) framework to characterize research gaps. While these two studies specifically focus on the use of systematic reviews to identify research gaps, other methods are being used, and further exploring these methods can optimize their definition, methodological scrutiny, and practice [4-14]. For example, scoping reviews and umbrella reviews are emerging methods for mapping and summarizing evidence, with an explicit aim of identifying research gaps in a broad area as compared with systematic reviews, which focus on answering a specific research question [15]. The aforementioned methods focus on the use of

For peer review only - http://bmjopen.bmj.com/site/about/guidelines.xhtml

secondary research methods to identify research gaps. However, a recent scoping review showed that other methods have been used to identify gaps, including primary and both primary and secondary research methods[1]. The scoping review confirmed what previous studies showed regarding a lack of consensus on what constitutes the best methodological approaches to identify research gaps, determine research priorities, and display research gaps or priorities [1],[5],[7]. Therefore, to better understand the different methods and on-going practices, we aimed to conduct a qualitative study to further explore more in-depth key stakeholder experiences on the methods used to identify gaps in health research.

Healthcare decisions for individual patients, public health policies, and clinical guidelines should be informed by the best available research evidence while taking into research gaps. Therefore, investigating experiences with practices/methods used to identify research gaps can inform explicit methodological approaches in identifying and describing research gaps and evidence-based practice. This investigation can enhance practices of different stakeholder groups (i.e., health professionals, commissioners, researchers, the public/patients and decision-makers) when addressing areas of uncertainty within the research problem and topic area[16]. Initiatives such as the James Lind Alliance (JLA), UK Database of Uncertainties about the Effects of Treatments, Cochrane Agenda and Priority Setting Methods Group and Evidence-based Research Network are some examples of existing efforts to identify and prioritize research gaps in health [1].

This study will be nested in a larger project aimed at developing methodological guidance for identifying gaps in health research. The first step in the project was a scoping review describing methods used to identify, prioritize and display gaps in health research. The scoping review mapped evidence on different definitions reported for the term "research gap" as well as methods used to identify research gaps and determine research priorities and display research gaps or research priorities. The scoping review reported different definitions and methodological approaches used for the topic.[1]. The second step is the qualitative study described in this protocol. The aim of the study is to further investigate perspectives and practices of key stakeholders identified in the scoping review (public/patients, researchers, clinicians, clinical guideline developers, public health professionals,

For peer review only - http://bmjopen.bmj.com/site/about/guidelines.xhtml

policy makers and funders). The final step will be an integration and overview combining findings from both studies to inform the methodological guidance on identifying research gaps.

The specific objectives of the study are to 1) investigate key stakeholders' knowledge, perceptions and experiences on defining research gaps and 2) characterize methods/practices used for identifying and displaying gaps in health research.

METHODS AND ANALYSIS

Qualitative study design

This study is an exploratory qualitative study using semi-structured interviews. This method will provide in-depth insight into key stakeholders' perspectives, experiences, and practices with defining, identifying and displaying research gaps. Investigating perspectives of different key stakeholders will ensure that the issue is not explored through one lens but rather a variety of lenses. This will allow for multiple facets of research gaps including definitions, methodological approaches/practices to identify and display gaps to be revealed and understood better [17].

Study sample and recruitment

The study sample will include the following stakeholder groups (i.e., researchers, funders, clinicians, clinical guideline developers, public health professionals, commissioners, public/patients and policymakers). The stakeholder groups will be organized in three main categories focusing on the use of evidence to inform health research, health practice or policymaking. These categories (policy, practice and research) are determined from the scoping review findings [1]. Therefore, the conception and design of the study and selection of participants are directly linked to the scoping review. More information and examples of organizations are given in Table 1. The identification of study participants will be recruited via contacts and organizations identified in the scoping review, existing professional networks (e.g., H2020 Project MiRoR) and contacts from conference attendance (e.g., Evidence Live and Cochrane Colloquium).

For peer review only - http://bmjopen.bmj.com/site/about/guidelines.xhtml

This study will also include patients or members of the public as key informants, which will allow for better understanding participants' perceived needs and priorities in identifying research gaps to make informed health decisions.

We will use purposive sampling to ensure that the perspectives of all the identified stakeholder groups are represented. Purposeful sampling is widely used in qualitative research for identifying and selecting information-rich cases, and in this study further elaboration of the term research gap is needed to better understand the context of the research gaps and methods/practices used to identify and display the research gaps [18, 19].

We anticipate performing about 14 to 28 interviews. This number of interviews will provide for data saturation needed (i.e., the point when new data do not add to a better understanding of the studied phenomenon but rather repeat what was previously expressed [20]) and also obtain a scope of responses from each stakeholder group. This estimation of interview participants is based on a study including 60 interviews that showed saturation with 12 interviews, with broader themes apparent after only 6 interviews [21]. The authors noted that factors such as heterogeneity of the sample affect how many interviews are required but concluded that to understand common perceptions and experiences among a group of relatively homogeneous individuals, 12 interviews should suffice[21]. Another study, after examining 25 in-depth interviews, found code saturation reached after 9 interviews, with the range of thematic issues identified; the authors proposed 16 to 24 interviews to reach saturation (i.e., a richly textured understanding of issues [22]). Therefore, we aim to gather 14 to 28 interviews for our three main categories (health policy and practice and informing future research).

Saturation will be guided by the seven parameters identified by Hennink et al., [22, 23] including the study purpose, population, sampling strategy, data quality, type of codes, code book and saturation goal and focus retrieved from the study. Each of these parameters will be considered throughout the study.

For peer review only - http://bmjopen.bmj.com/site/about/guidelines.xhtml

170 Table 1. Key informants

Categories	Key informants	Examples of organizations	Expected number of interviews
Health research	Researchers	Research institutes/universities (lead researchers)<br>Cochrane Priority Setting Methods Group<br>Knowledge synthesis research groups	2–4
	Funding bodies	UK National Institute for Health Research<br>European Union	2–4
Health practice	Clinicians	UK National Health Service	2–4
	Clinical guideline developers	UK National Institute for Health and Care Excellence	2–4
	Public health professionals, Commissioners	National public health bodies (e.g., Public Health England)	2–4
	Public/patients	James Lind Alliance<br>Patient forums/groups	2–4
Policymaking	Policymakers	UK National Health Service<br>Ministry of Health officials	2–4

171

172 **Data collection and recording**

173 Semi-structured interviews will be used for this study. The main reason for selecting semi-structured
174 interviews is to allow for specific areas to be addressed while giving the interviewees the opportunity
175 to reflect on their experiences and perspectives related to defining, identifying, and presenting
176 research gaps that are relevant to them and may not have been explored or anticipated by the
177 researcher(s) [24].

178 We will conduct interviews in-person and using teleconference, according to the participant's
179 availability and preference. In-person interviews will be conducted primarily with participants
180 residing or reachable in London, UK, and other participants will be interviewed via teleconference
181 (see Appendix 1 for the interview guide for both the in-person and teleconference interviews). The
182 interviews will be recorded on a digital recorder for face-to-face interviews and electronically for
183 teleconference interviews.

For peer review only

For peer review only - http://bmjopen.bmj.com/site/about/guidelines.xhtml

The guide was developed by focusing on exploring key stakeholder perspectives and experiences with the following key areas:

1) Participant background information
2) Definitions of research gaps
3) Knowledge, perceptions and experiences on methods/practices used to identify and display gaps in health research to inform further research, practice and policy

These three domains were developed with information from the scoping review to guide the questions. The interview topic guide will be piloted to before data collection. It will also be adapted according to key stakeholder groups to ensure it is meaningful to their background and to gather more relevant information based on their experiences and knowledge [25].

The semi-structured interview guide consists of two levels of questions: main themes and follow-up questions. The main themes cover the general content of the research gaps aimed to encourage participants to speak freely about their perceptions, experiences, and practices. Follow-up questions are prompts and probes aimed at following respondents' answers and investigating the raised issues more in-depth. The interview guide covers the main topics of the study, providing a focused structure for the discussion during the interviews. However, it does not need to be strictly followed — the main focus is on providing a setting that encourages respondents to share their perceptions and experiences with research gaps as thoroughly as possible within the constraints of our study aims [26].

All interviews will be transcribed verbatim and anonymised. The lead researcher (LN) will transcribe two interviews to help inform the analytic process, and the other audio files will be transcribed by a professional transcription agency licensed from the University of Liverpool.

Data analysis

We will use analytical categories to describe and explain definitions, experiences and practices reported among the groups of participants. All data relevant to each category (describing research gaps, experience with identifying and displaying research gaps) will be identified and examined to ensure that each data item is checked accordingly.

For peer review only - http://bmjopen.bmj.com/site/about/guidelines.xhtml

Our approach is based on the thematic analysis outlined by Braun and Clarke [27]. The steps include the following: 1) Transcription and checking transcripts with recordings for accuracy; 2) open coding from interview responses will be performed by two researchers independently (LN and DH); 3) agreement of initial codes will be discussed among the researchers and an initial codebook will be developed; 4) the code structure will be used for analysing the remaining responses with openness to including new codes and refining existing ones; and 5) themes and subthemes will be identified from the final code structure, and their relationships will be represented by using a thematic map [27].

The initial coding framework for our analysis will start from broad categories identified in the previous scoping review, in which the interviews were structured. Within these broad categories (e.g., definitions, practices to identify gaps, practices to display gaps), analytic categories will be inductively derived from the data. In this sense, our approach includes both top-down and bottom-up development of analytic categories and themes.

Trustworthiness during thematic data analysis will be ensured by storing raw data systematically, documenting detailed notes about the development and hierarchies of concepts and themes, establishing consensus on themes, providing detailed descriptions of context, and describing the process of coding and analysis [8, 9]. NVivo 12 Pro, a qualitative data analysis software, will be used for data management and analysis.

Ensuring study quality

To further ensure rigour and trustworthiness, this study will be guided by Guba and Lincoln's concepts for defining and investigating quality in qualitative research that can be considered parallel to quantitative research concepts of validity and reliability [23, 28, 29]. The concepts include credibility, transferability, dependability, confirmability, audit trails and reflectivity. They are interrelated, and thinking through them from the onset and incorporating them in a study will improve the study rigor.

Credibility is the interaction between respondents' views and the researcher's perspectives of the views [30]. One of the ways we will ensure credibility is by planning debriefing sessions between the

For peer review only - http://bmjopen.bmj.com/site/about/guidelines.xhtml

lead researcher (LN) and senior researcher (DH) to provide a sounding board for LN on elaborating different study ideas and interpretations [6]. Transferability refers to the generalizability of the study findings [17, 30]. We aim to address this criterion by reporting the rich background and context descriptions of our study findings from the key stakeholders. Dependability is related to whether the research questions are clear and logically connected to the research purpose and design [30]. We aim to achieve this by first drafting this protocol to guide our study and future studies with a similar purpose. Confirmability has been related to objectivity or neutrality aimed to establish that the data and interpretations of the findings are not figments of the inquirer's imagination but are clearly derived from the data, establishing that data collection and interpretations of the study are clearly deliberated from the data and not misinterpreted[30]. We aim to address confirmability by documenting the justification of methodological and analytical choices to illustrate how the data were derived in relation to the study objectives and transparently describing the research steps taken from the start of the project to the development and reporting of the findings. Records of the research path will be kept throughout the study as well de-briefing sessions held between the main researcher (LN) and senior researcher (DH). Finally, reflexivity includes examining one's own conceptual lens, explicit and implicit assumptions, preconceptions and values and how these affect research decisions in all phases of qualitative studies. Reflexivity will be assured by maintaining a reflexive journal to document the different thoughts and reflections throughout the study process[30] and discussing any emerging issues between the main researcher (LN) and senior researcher (DH).

Patient or public involvement

There is no patient or public involvement in the design or conduct of this planned study.

DISCUSSION

This study will provide insights into issues related to defining research gaps and methods used to identify and display gaps in health research from perspectives of key stakeholders involved in the process. This is a follow-up study of a wider project; the first study was a scoping review exploring methods used to identify and display research gaps reported in scientific publications[1]. The scoping review showed variation and ambiguity in how research gaps are described as well as the methods

For peer review only - http://bmjopen.bmj.com/site/about/guidelines.xhtml

used to identify and prioritize research gaps. Several of the articles described the development of a framework or tool for identifying and prioritizing research gaps and applying it to a specific topic area as an example for application [1-3, 31]. There were no evaluations of reproducibility of the method/frameworks identified in the scoping review [1, 3]. Furthermore, despite articles highlighting the existence of research gaps in their study, very few specifically described the gaps and the causes or the method of identification, so fully understanding the relevance and importance of the research gap to adequately address it is difficult. Our scoping review also primarily found the use of secondary research methods such as systematic reviews and scoping reviews as the most commonly used method to identify gaps; although other methods were identified, they were inadequately described. The scoping review also showed that besides researchers, different audiences including clinicians, policy makers, funders and patients or the public can benefit from understanding gaps and methods/practices on how to identify and display gaps in health research. This qualitative study aims to go beyond the scientific literature in describing, identifying and displaying gaps in health research and directly talk to people about their understanding and practices. Given the nature of this topic area that is not fully explored, there is a need to investigate real practices to be able to develop methodological guidance taking into consideration the existing literature and on-going practices.

This study has some limitations; one is not including patients/public in designing the study. This would have benefited the study design by including patients/public perspectives to be able to improve the importance and relevance of the findings for this population. This study is also primarily focused on key organizations found in the scoping review that are mainly UK-based, which may limit perspectives and experiences on mapping research gaps. To address this situation, we aim to identify other relevant potential organizations and interview participants outside the United Kingdom. One of the main strengths of the study is improving the definition of research gaps, and subsequently improving accurate reporting of research gaps to clearly elucidate the characteristics, which can help in making evidence-based decisions. For example, making a decision based on a research gap contributing to lack of primary research on a specific health problem can differ from a research gap related to lack of secondary research summarizing the research. Hence, all these factors regarding research gaps need to be highlighted if they are known and made explicit when disseminating and

For peer review only - http://bmjopen.bmj.com/site/about/guidelines.xhtml

communicating research. Additionally, providing more information on what the gap represents may inform users of evidence on more specific information about the research gap and how it can be addressed more accurately. We anticipate that this study will advance efforts in research and practice on this topic area.

ETHICS and DISSEMINATION

Informed consent will be obtained in accordance with the University of Liverpool Ethics Committee board requirements. Verbal consent will be sought for phone interviews and written consent for in-person interviews. Confidentiality and data protection will be ensured in accordance with the University of Liverpool Ethics Committee board. All participant information will be anonymized, and hard-copy data will be stored in a locked unit. Soft-copy material will be stored in a password-protected file. Upon completion of the study and publication of the study results, all study material will be stored and disposed of according to the rules and regulations of the University of Liverpool. The study protocol will be stored in the data repository Zenodo. The research has obtained ethical approval from the University of Liverpool, UK.

At the end of this research project, the results will be presented at conferences and relevant meetings (e.g., H2020 Project MiRoR). They will also be published in a peer-reviewed journal and as part of a doctoral thesis of the PhD fellow (LN) as well as in professional and lay magazines and presented in workshops at professional events for stakeholder groups and as online materials with good practice examples.

Acknowledgements

We acknowledge the feed-back and support of Daniela Lai and Cristian R. Montenegro in providing feedback on the interview guide. We thank Laura Smales (BioMedEditing, Toronto, ON) for editing the manuscript.

For peer review only

Authors' contributions

LN and DH conceived the study with guidance and feedback from RP and CTM. All authors read and approved the final manuscript.

Funding

This project is a part of a MiRoR (Methods in Research on Research)-funded PhD being undertaken by LN. MiRoR received funding from the European Union's Horizon 2020 research and innovation programme under a Marie Sklodowska-Curie grant (agreement no. 676207).

Conflict of interest

The authors declare no conflicts of interest.

For peer review only

For peer review only - http://bmjopen.bmj.com/site/about/guidelines.xhtml

References

1. Nyanchoka L, Tudur-Smith C, Thu VN, Iversen V, Tricco AC, Porcher R: **A scoping review describes methods used to identify, prioritize and display gaps in health research**. *Journal of Clinical Epidemiology* 2019, **109**:99-110.

2. Robinson KA, Saldanha IJ, McKoy NA: **Development of a framework to identify research gaps from systematic reviews**. *J Clin Epidemiol* 2011, **64**(12):1325-1330.

3. Carey T, Yon A, Beadles C, Wines R: **Prioritizing future research through examination of research gaps in systematic reviews**. *Prepared for the Patient-Centered Outcomes Research Institute* 2012.

4. Whittemore R, Chao A, Jang M, Minges KE, Park C: **Methods for knowledge synthesis: An overview**. *Heart & Lung: The Journal of Acute and Critical Care* 2014, **43**(5):453-461.

5. Kastner M, Antony J, Soobiah C, Straus SE, Tricco AC: **Conceptual recommendations for selecting the most appropriate knowledge synthesis method to answer research questions related to complex evidence**. *Journal of Clinical Epidemiology* 2016, **73**:43-49.

6. Manson H: **Systematic reviews are not enough: policymakers need a greater variety of synthesized evidence**. *Journal of Clinical Epidemiology* 2016, **73**:11-14.

7. Perrier L, Lightfoot D, Kealey MR, Straus SE, Tricco AC: **Knowledge synthesis research: a bibliometric analysis**. *Journal of Clinical Epidemiology* 2016, **73**:50-57.

8. Pluye P, Hong QN, Bush PL, Vedel I: **Opening-up the definition of systematic literature review: the plurality of worldviews, methodologies and methods for reviews and syntheses**. *Journal of Clinical Epidemiology* 2016, **73**:2-5.

9. Sales A: **Generating and using evidence: reflections from the perspective of implementation**. *Journal of Clinical Epidemiology* 2016, **73**:6-7.

10. Straus SE, Kastner M, Soobiah C, Antony J, Tricco AC: **Introduction: Engaging researchers on developing, using, and improving knowledge synthesis methods: a series of articles describing the results of a scoping review on emerging knowledge synthesis methods**. *Journal of Clinical Epidemiology* 2016, **73**:15-18.

11. Tricco AC, Antony J, Soobiah C, Kastner M, Cogo E, MacDonald H, D'Souza J, Hui W, Straus SE: **Knowledge synthesis methods for generating or refining theory: a scoping review reveals that little guidance is available**. *Journal of Clinical Epidemiology* 2016, **73**:36-42.

12. Tricco AC, Antony J, Soobiah C, Kastner M, MacDonald H, Cogo E, Lillie E, Tran J, Straus SE: **Knowledge synthesis methods for integrating qualitative and quantitative data: a scoping review reveals poor operationalization of the methodological steps**. *Journal of Clinical Epidemiology* 2016, **73**:29-35.

13. Tricco AC, Soobiah C, Antony J, Cogo E, MacDonald H, Lillie E, Tran J, D'Souza J, Hui W, Perrier L *et al*: **A scoping review identifies multiple emerging knowledge synthesis methods, but few studies operationalize the method**. *Journal of Clinical Epidemiology* 2016, **73**:19-28.

14. Wong G: **Knowledge synthesis approaches–spoilt for choice?** *Journal of Clinical Epidemiology* 2016, **73**:8-10.

For peer review only - http://bmjopen.bmj.com/site/about/guidelines.xhtml

15. Birte S, Martina V, Ami B, Jennifer S, Marie G: **Evidence & Gap Maps: A tool for promoting evidence informed policy and strategic research agendas**. *Journal of Clinical Epidemiology* 2016, **79**:120.

16. Bouma GD, Ling R: *The research process*: Oxford University Press, USA; 2004.

17. Yin RK: **Case study research: Design and methods (applied social research methods)**. *London and Singapore: Sage* 2009.

18. Setia MS: **Methodology Series Module 5: Sampling Strategies**. *Indian journal of dermatology* 2016, **61**(5):505-509.

19. Patton MQ: *Qualitative evaluation and research methods*: SAGE Publications, inc; 1990.

20. Saunders B, Sim J, Kingstone T, Baker S, Waterfield J, Bartlam B, Burroughs H, Jinks C: **Saturation in qualitative research: exploring its conceptualization and operationalization**. *Quality & Quantity* 2017.

21. Guest G, Bunce A, Johnson L: **How Many Interviews Are Enough?: An Experiment with Data Saturation and Variability**. *Field Methods* 2006, **18**(1):59-82.

22. Hennink MM, Kaiser BN, Marconi VC: **Code Saturation Versus Meaning Saturation: How Many Interviews Are Enough?** *Qualitative Health Research* 2016, **27**(4):591-608.

23. Shenton A: *Strategies for Ensuring Trustworthiness in Qualitative Research Projects*. *Volume 22*; 2004.

24. Britten N: **Qualitative interviews in medical research**. *BMJ* 1995, **311**(6999):251-253.

25. Kvale S, Brinkmann S: *Interviews: Learning the craft of qualitative research interviewing*: Sage; 2009.

26. Gill P, Stewart K, Treasure E, Chadwick B: **Methods of data collection in qualitative research: interviews and focus groups**. *Br Dent J* 2008, **204**(6):291-295.

27. Braun V, Clarke V: **Using thematic analysis in psychology**. *Qualitative Research in Psychology* 2006, **3**(2):77-101.

28. Guba EG, and Yvonna S. Lincoln: *Naturalistic inquiry*; 1985.

29. Glonti K, Hren D: **Editors' perspectives on the peer-review process in biomedical journals: protocol for a qualitative study**. *BMJ Open* 2018, **8**(10):e020568.

30. Tobin GA, Begley CM: **Methodological rigour within a qualitative framework**. *J Adv Nurs* 2004, **48**(4):388-396.

31. Robinson KA, Saldanha IJ, McKoy NA: **Identification of research gaps from evidence-based guidelines: a pilot study in cystic fibrosis**. *Int J Technol Assess Health Care* 2011, **27**(3):247-252.

For peer review only

For peer review only - http://bmjopen.bmj.com/site/about/guidelines.xhtml

Key stakeholders' perspectives and experiences with defining, identifying and displaying gaps in health research: a qualitative study protocol

Appendix 1: Semi-structured interview guide

Date:	Interviewer:	Archival #:	
In person:	Teleconference:	Start Time:	End Time:

Background?

1. Tell me a little about your work, and what you do?
   What does it involve?
2. Experience with using evidence for decision-making in health choices, policymaking, prioritizing research or funding projects?
3. How did you go about making the decision when the evidence was missing, insufficient or inadequate?

Defining research gaps

4. How would you describe the term "research gaps" in your own words?
   Probe based on participant (Researcher, Policy maker, Funder, Health Professional or Public/Patient)
   - *(Research) Can you walk me through how you use evidence to inform future research/research topics?*
   - *(Policy Makers) Can you walk me through how you use research to influence policies?*
   - *(Funders) Can you walk me through how you use research to determine which project to fund?*
   - *(Health Professionals) Can you walk me through how you use research to inform your practice as a health provider?*
   - *(Public/Patients) Can you walk me through how you use research to inform your health decisions?*
     - ✓ What are your thoughts on the importance of identifying research gaps?
     - ✓ What are your thoughts on the causes of research gaps?

Experiences, knowledge and perceived needs with methods used to identify research gaps

5. Could you talk about your views/any experience you have in identifying research gaps?
   - *(Research) For example, if you need to apply for funding, how would you select the study?*
   - *(Policy Makers) For example, if you work in developing policies?*
   - *(Funders) or example, if you need to fund projects, how do you determine which ones to fund?*
   - *(Health professionals) For example, in making decisions between treatment choices in your practice where there is uncertainty?*
   - *(Public/Patients) For example, when making health decisions where there is uncertainty?*

6. Could you tell me more specifically about the methods you used to identify research gaps?
   - ✓ What are some of the strengths of the method(s)/practices you used?
   - ✓ What are some of the challenges you experienced using the method(s) /practices?
7. Looking back on your experience using methods to identify research gaps, what is needed to improve the methods you used to identify research gaps?

Experiences, knowledge and perceived needs with methods used to display/present research gaps

8. Could you describe any experience you have in displaying/presenting research gaps?
9. Could you tell me more about the method(s) you used to display/present research gaps?
   - ✓ What are some of the strengths of the method(s) you used for displaying research gaps?
   - ✓ What are some of the challenges you experienced?
10. Please share any reflection on what you feel is needed to improve the methods you used to display/present research gaps?

General follow-up questions

11. Any additional thoughts you would like to share?

For peer review only - http://bmjopen.bmj.com/site/about/guidelines.xhtml

Key stakeholders' perspectives and experiences with defining, identifying and displaying gaps in health research: a qualitative study protocol

Appendix 2: Participant Information Sheet

Experiences with Methods for Identifying and Displaying Research Gaps

We invite you to take part in our research study. Before you decide whether to participate, you should understand why the research is being done and what it will involve. Please take your time to read the following information carefully and feel free to ask if you need more information or if there is anything that you do not understand. Please also feel free to discuss this with your friends, relatives and anyone else you wish.

What is the purpose of the study?

This study aims to explore the experiences of key stakeholders, including **the public, patients, researchers, clinicians, clinical guideline developers, public health professionals, policymakers and funders**, with methods for identifying and displaying research gaps, to inform **health choices, health practice, future research, policy or funding**. This study aims to help in better understanding the methods used to identify and display research gaps. The overall topic area on methods to identify and display gaps is still not well established, particularly because of no standard definition for the term "research gaps"; therefore a study to better understand the context, as well as the interactions of the factors such as alternative definitions, different audiences and methods used to identify gaps is important to improve our understanding of the audience's needs and the strengths and limitations of methods.

Why have I been chosen to take part?

You have been asked to take part because you are or have been involved in using research, producing research and/or communicating research. Your insight and experience with any methods you have used to identify and display research gaps will be highly appreciated to further guide this topic area.

Do I have to take part?

It is completely up to you whether or not you agree to take part in the study. If you do decide to take part, you will be asked to sign a consent form. If you decide to take part but then change your mind, you are free to do so at any time without giving a reason.

What will happen if I take part?

You will be asked to take part in an interview with a researcher, Linda Nyanchoka, about your experience with and your views of methods for identifying and display research gaps. The interviews will last approximately 20 to 40 minutes or as long as you would like to talk about your experience. With your permission, the interview will be audio-recorded. You can stop the interview at any time, and you do not have to answer a particular question if you don't want to.

Where will the interview take place?

The interview will take place in person at a specific location or over the phone. Participants in the UK have the option of an in-person or teleconference interview, and all other participants will have teleconference interviews at a date and time that is convenient for them.

Are there any risks in taking part?

We do not expect any risks or discomfort associated with this research study. However, if you feel uncomfortable, you can stop the interview at any time, without giving a reason.

For peer review only - http://bmjopen.bmj.com/site/about/guidelines.xhtml

Key stakeholders' perspectives and experiences with defining, identifying and displaying gaps in health research: a qualitative study protocol

Are there any benefits in taking part?

You will be helping develop our understanding of research gaps and methods for identifying and displaying research gaps.

Will my participation be kept confidential?

All the information you give us will be kept strictly confidential. The procedures for handling, processing, storing and destroying the data will comply with the Data Protection Act of 1998.

This means that only the researchers will see what you have said. The audio-recording of your interview will be identified by a code number only. These audio-recordings will be transcribed, and identifying details such as place names and people's names will be removed from the transcripts. We will use quotes from the interviews in the write-up of the study but will ensure that no one can be identified from these quotes.

At the end of the study, the research data, including consent forms, anonymised interview transcripts, field notes and your contact details, will be kept in locked filling cabinets and/or password-protected university computers for up to 10 years.

What will happen to the results of the study?

After the study has finished, the results will be written up as part of the PhD research thesis of Linda Nyanchoka and submitted for examination. The results will also be submitted for publication in an academic journal and presented at conferences.

If you would like to receive a copy of the findings, please let us know by using the contact information provided and we will happily provide you with one.

What will happen if I want to stop taking part?

If you decide at any point that you no longer wish to be part of the study, then you can withdraw without giving a reason. You can also ask for your data to be removed from the study and destroyed.

What if I am unhappy or if there is a problem?

If you are unhappy or if there is a problem, please feel free to let us know by contacting the lead researcher, Linda Nyanchoka, at the University of Liverpool (+33 75 34 29 417; L.Nyanchoka@liverpool.ac.uk). Linda will try to help or put you in touch with someone who can.

If you remain unhappy or have a complaint that you feel you cannot communicate to us, you should contact the Research Governance Officer at the University of Liverpool (0151 794 8290; ethics@liv.ac.uk). When contacting the Research Governance Officer, please provide the name or a description of the study (so that it can be identified), the researcher(s) involved, and the details of the complaint you wish to make.

Who is funding the research?

This research is funded by the European Union's Horizon 2020 research and innovation programme under the Marie Sklodowska-Curie grant (agreement no. 676207). If you want to find out more about the funding body, please contact https://ec.europa.eu/programmes/horizon2020/.

Who is doing this research?

The research and interviews will be conducted by Linda Nyanchoka, a Marie Curie Research Fellow at the University of Liverpool, UK.

For peer review only - http://bmjopen.bmj.com/site/about/guidelines.xhtml

Key stakeholders' perspectives and experiences with defining, identifying and displaying gaps in health research: a qualitative study protocol

How can I find out more?

You can get in touch with Linda Nyanchoka, who will be happy to answer any questions you might have:

Department of Biostatistics,

Institute of Translational Medicine

Block F/Waterhouse Buidling,

University of Liverpool,

Liverpool

L69 3BX

Teleconference no.: +33 75 34 29 417

Email address: lnyanchoka@gmail.com or L.Nyanchoka@liverpool.ac.uk

Thank you for taking the time to read this document.

This information sheet is for you to keep

For peer review only - http://bmjopen.bmj.com/site/about/guidelines.xhtml

Key stakeholders' perspectives and experiences with defining, identifying and displaying gaps in health research: a qualitative study protocol

Appendix 3: Participant consent form

Researcher: Linda Nyanchoka

Please initial box

1. I confirm that I have read and have understood the information sheet dated **[ ]** for the above study. I have had the opportunity to consider the information, ask questions and have had these answered satisfactorily. ☐

2. I understand that my participation is voluntary and that I am free to withdraw at any time without giving any reason, without my rights being affected. ☐

3. I understand that, under the Data Protection Act 1998, I can at any time ask for access to the information I provide and I can also request the destruction of that information if I wish. ☐

4. I agree for the data I provide to be archived at The University of Liverpool. I understand that other authorised researchers will have access to this data only if they agree to preserve the confidentiality of the information as requested in this form. ☐

5. I agree to take part in the above study. ☐

______________________	__________	______________________
Participant name	Date	Signature
______________________	__________	______________________
Name of person taking consent	Date	Signature
______________________	__________	______________________
Researcher	Date	Signature

For peer review only

Key stakeholders’ perspectives and experiences with defining, identifying and displaying gaps in health research: a qualitative study protocol

Principal Investigator

Catrin Tudur-Smith
University of Liverpool
Biostatistics Department
Block F Waterhouse Building
1-5 Brownlow
Liverpool L69 3GL

Tel: +44 (0)151 794 4059
Email: cat1@liverpool.ac.uk

Student Investigator

Linda Nyanchoka
University of Liverpool
Biostatistics Department
Block F Waterhouse Building
1-5 Brownlow Street
Liverpool L69 3GL

Tel: +33 75 34 29 417
Email: L.Nyanchoka@liverpool.ac.uk

The information you have submitted will be published as a report; please indicate whether you would like to receive a copy. ☐

I understand that confidentiality and anonymity will be maintained and it will not be possible to identify me in any publications ☐

I agree for the data collected from me to be used in future research and understand that any such use of identifiable data would be reviewed and approved by a research ethics committee. ☐

I understand and agree that my participation will be audio recorded and I am aware of and consent to your use of these recordings for the following purposes: meeting research aims and goals in exploring methods used to identify and display research gaps. ☐

I understand that the information collected about me will be used to support other research in the future, and may be shared anonymously with other researchers. ☐

I would like my name used and I understand and agree that what I have said or written as part of this study will be used in reports, publications and other research outputs so that anything I have contributed to this project can be recognised. ☐

I understand that my responses will be kept strictly confidential. I give permission for members of the research team to have access to my anonymised responses. I understand that my name will not be linked with the research materials, and I will not be identified or identifiable in the report or reports that result from the research. ☐

For peer review only

Key stakeholders' perspectives and experiences with defining, identifying and displaying gaps in health research: a qualitative study protocol

I understand and agree that once I submit my data it will become anonymised and I will therefore no longer be able to withdraw my data. ☐

I understand that the fully anonymised data will be held securely at the University of Liverpool and I can request access to the data collected, and/or request that the data is destroyed at any time until the data is submitted for publication. ☐

I understand that other authorised researchers may use my words in publications, reports, webpages, and other research outputs, only if they agree to preserve the confidentiality of the information as requested in this form. ☐

For peer review only

For peer review only - http://bmjopen.bmj.com/site/about/guidelines.xhtml

Key stakeholders' perspectives and experiences with defining, identifying and displaying gaps in health research: a qualitative study protocol

Appendix 4: Participant Teleconference Consent Form

Teleconference: Oral Consent Example Script:

Hello, I am Linda Nyanchoka, a PhD student from the University of Liverpool. I will be talking to you about my research project on defining research gaps and on methods to identify and display research gaps in health. Additional information is on the information sheet you have received.

Are you still interested in taking part in the project? *[Await confirmation]*. Now I'd like to confirm some of the details of the project to make sure you are clear about what's involved for you:

- We do not expect any risks or discomfort associated in this research study. However, if you feel uncomfortable, you can stop the interview at any time, without giving a reason.
- You do not have to say yes to take part; you can ask me any questions you want before or during the interview; you can also withdraw at any stage without giving a reason and without any negative consequences.
- You do not have to answer any questions that you do not wish to.
- You are aware that a University of Liverpool Research Ethics committee has approved this research project; for further information email me at L.Nyanchoka@liverpool.ac.uk
- I may use brief quotes of what you say during the interview in the write-up of this study, but they will remain anonymous.
- I will safely store your data electronically in encrypted, secure files. All identifiable data will be destroyed at the end of the study.
- I will audio-record you unless you say that I can't.
- Are you still willing to take part?
  Do you give your permission for me to re-contact you to clarify information?

[*Await confirmation]* So if you're happy with all of that, and have no more questions, let's start.

Researcher: Linda Nyanchoka

Participant:

Date:

Time:

For peer review only - http://bmjopen.bmj.com/site/about/guidelines.xhtml

BMJ Open

Key stakeholders' perspectives and experiences with defining, identifying and displaying gaps in health research: a qualitative study protocol

Journal:	*BMJ Open*
Manuscript ID	bmjopen-2018-027926.R2
Article Type:	Protocol
Date Submitted by the Author:	27-Jun-2019
Complete List of Authors:	Nyanchoka, Linda; Université Paris Descartes, ; University of Liverpool, Institute of Translational Medicine<br>Tudur-Smith, Catrin; University of Liverpool, Biostatistics<br>Porcher, Raphaël; Inserm U1153<br>Hren, Darko; University of Split, School of Humanities and Social Sciences
**Primary Subject Heading**:	Qualitative research
Secondary Subject Heading:	Research methods
Keywords:	Research Uncertainties, Identifying Research Gaps, Displaying Research Gaps, Evidence Synthesis, Evidence-based Decision-making, QUALITATIVE RESEARCH

SCHOLARONE™
Manuscripts

For peer review only - http://bmjopen.bmj.com/site/about/guidelines.xhtml

Title

Key stakeholders' perspectives and experiences with defining, identifying and displaying gaps in health research: a qualitative study protocol

Authors

Linda Nyanchoka[1, 2, 3], Catrin Tudur-Smith[3], Raphaël Porcher[1, 2, 4], Darko Hren[5]

Affiliations

1. Université Paris Descartes, Sorbonne Paris Cité, Faculté de Médecine, Paris, France
2. INSERM, UMR1153, Epidemiology and Statistics Sorbonne Paris Cité Research Center (CRESS), Team METHODS, Paris, France
3. University of Liverpool, Institute of Translational Medicine, Liverpool, United Kingdom
4. Assistance Publique des Hôpitaux de Paris (AP-HP), Hôpital Hôtel-Dieu, Center for Clinical Epidemiology, Paris, France
5. University of Split, Department of Psychology, Faculty of Humanities and Social Sciences, Split, Croatia

Keywords

Evidence Synthesis; Research Gaps; Research Priorities; Identifying Research gaps; Displaying Research Gaps; Evidence-based decision-making; Qualitative Study

Correspondence

Linda Nyanchoka lnyanchoka@gmail.com

1. Université Paris Descartes, Sorbonne Paris Cité, Faculté de Médecine, Paris, France
2. INSERM, UMR1153, Epidemiology and Statistics Sorbonne Paris Cité Research Center (CRESS), Team METHODS, Paris, France
3. University of Liverpool, Institute of Translational Medicine, Liverpool, United Kingdom

Word Count: Abstract: 282 Manuscript: 3462

For peer review only

Abstract

Introduction

Identifying research gaps can inform the design and conduct of health research, practice and policies by informing the current body of evidence. Audiences including researchers, clinical guideline developers, clinicians, policymakers, research regulatory bodies, funders and patients/the public can also benefit from understanding the status of research and research gaps to make informed choices. This study aims to explore how key informants define research gaps and characterize methods/practices used to identify and display gaps in health research to inform future research practice and policies.

Methods and analysis

This is an exploratory qualitative study using semi-structured in-depth interviews. The participants will be recruited by purposive sampling from initiatives and organizations previously identified in a scoping review on methods to identify, prioritize and display gaps in health research. We anticipate performing up to 28 interviews with the different key informant groups who are involved in using evidence to inform health policy, practice, and research. Interviews will be thematically analysed as outlined by Braun and Clarke. The qualitative data-analysis software NVivo 12 Pro will be used to aid data management and analysis.

Discussion

This is the protocol for a follow-up study that aims to complement and enrich the findings of the scoping review on methods to identify, prioritize and display gaps in health research. The overall project aims to develop methodological guidance for describing, identifying and displaying gaps in health research.

Ethics and dissemination

The research obtained ethical approval from the University of Liverpool, UK. The findings will be disseminated via conferences, meetings (organized by the Methods in Research on Research project),

For peer review only - http://bmjopen.bmj.com/site/about/guidelines.xhtml

peer-reviewed publications and lay magazines because the study participants will include the public/patients.

Strengths and limitations

- The qualitative nature of this study provides an in-depth understanding of key informants' perspectives and experiences in describing, identifying and displaying gaps in health research.
- This study is embedded in a larger study aiming to develop methodological guidance to identify and display gaps in health research.
- This study would have benefited from including patient/public perspectives in designing the study to be able to improve the importance and relevance of the findings for this population.

For peer review only

For peer review only - http://bmjopen.bmj.com/site/about/guidelines.xhtml

BACKGROUND

Identifying research gaps can help inform the design and conduct of health research, practice and policies by providing a better understanding of the current body of evidence. The term “research gap” is not well defined, and its meaning can differ depending on the researcher and research context. A recent scoping review on methods used to identify, prioritize and display gaps in health research reported 12 different definitions related to gaps in health research (e.g., population, theoretical and methodology gaps), each describing research gaps differently [1]. This finding shows the ambiguity of the term “research gaps” and the different practices it may be related to.

As a basis for further exploring and understanding “research gaps”, we start from the definition given by the National Collaborating Center for Methods and Tools (NCCMT) in Canada based on the work of Robinson et al., whereby a research gap is defined as a topic or area for which missing or insufficient information limits the ability to reach a conclusion for a question [2]. Given the different meanings and definitions of research gaps found in the scoping review [1], we consider it important to further explore definitions rather than just adopt or modify the NCCMT definition. Clearly defining the type of research gap can help determine how to better identify, characterize, prioritize and address research gaps.

Different methods for identifying research gaps have been reported; for example, scoping reviews and umbrella reviews are emerging methods for mapping and summarizing evidence. These methods have an explicit aim of identifying research gaps in a broad area as compared with systematic reviews, which focus on answering a specific research question [3-7]. Robinson et al. developed a framework using systematic reviews to identify research gaps [2] in which they classified the reasons for the existence of research gaps and used the Population, Intervention, Comparison, Outcome and Setting (PICOS) process to characterize them. Scoping, umbrella and systematic reviews are reported to specifically identify research gaps, but other methods are being used, and further exploring these methods can optimize their definition, methodological scrutiny, and practice [8-18]. Furthermore, the aforementioned methods focus on the use of secondary research methods to identify research gaps. However, a recent scoping review showed that other methods have been used to identify gaps,

For peer review only - http://bmjopen.bmj.com/site/about/guidelines.xhtml

including primary and both primary and secondary research methods [1]. The scoping review showed a lack of consensus on what constitutes the best methodological approaches to identify research gaps, determine research priorities, and display research gaps or priorities [1, 5, 7]. Therefore, to better understand the different methods and ongoing practices, we aimed to conduct a qualitative study to further explore more in-depth key stakeholder experiences in describing research gaps and the methods used to identify and display gaps in health research.

This study is part of larger ongoing efforts to avoid waste in producing and reporting research evidence, with a focus on the identification of research gaps[19]. Healthcare decisions for individual patients, public health policies, and clinical guidelines should be informed by the best available research evidence while taking into consideration research gaps. Investigating experiences with practices/methods used to identify research gaps can inform explicit methodological approaches in identifying and describing research gaps. This investigation can enhance practices of different stakeholder groups (i.e., health professionals, commissioners, researchers, patients/the public and decision-makers) when addressing areas of uncertainty within the research problem and topic area[20]. Initiatives such as the James Lind Alliance (JLA), UK Database of Uncertainties about the Effects of Treatments, Cochrane Agenda and Priority Setting Methods Group, and Evidence-based Research Network are some examples of existing efforts to identify and prioritize research gaps in health [1].

This study is nested in a larger project aimed at developing methodological guidance for identifying gaps in health research. The first step in the project was a scoping review describing methods used to identify, prioritize and display gaps in health research in scientific literature. The scoping review mapped evidence on different definitions reported for the term “research gap” as well as methods used to identify research gaps and determine research priorities and display research gaps or research priorities [1]. The second step is the qualitative study described in this protocol. The aim of the study is to investigate the experience of key stakeholders (i.e., researchers, funders, clinicians, clinical guideline developers, public health professionals, commissioners, patients/the public and policymakers) with defining research gaps and practices/methods used to identify and display

For peer review only - http://bmjopen.bmj.com/site/about/guidelines.xhtml

research gaps. The final step will be an integration and overview combining findings from the scoping review and qualitative study to provide a comprehensive overview of methods used to identify and display research gaps. These study findings will be used to inform the methodological guidance on identifying research gaps.

The specific objectives of the study are to 1) investigate key stakeholders' knowledge, perceptions and experiences with defining research gaps and 2) characterize methods/practices used for identifying and displaying gaps in health research.

METHODS AND ANALYSIS

Qualitative study design

This study is an exploratory qualitative study using semi-structured interviews. This method will provide in-depth insight into key stakeholders' perspectives, experiences, and practices with defining, identifying and displaying research gaps. Investigating perspectives of different key stakeholders will ensure that the issue is not explored through one lens but rather a variety of lenses. This will allow for revealing and better understanding multiple facets of research gaps including definitions and methodological approaches/practices to identify and display gaps [21].

Study sample and recruitment

The study sample will include the following stakeholder groups (i.e., researchers, funders, clinicians, clinical guideline developers, public health professionals, commissioners, patients/the public and policymakers). The stakeholder groups will be organized in three main categories focusing on the use of evidence to inform health policy, health practice, and health research. These categories (policy, practice and research) are determined from the scoping review findings [1]. More information and examples of organizations are given in Table 1. Study participants will be recruited via contacts and organizations identified in the scoping review, relevant scientific publications, existing professional networks (e.g., H2020 Project MiRoR) and contacts from conference attendance (e.g., Evidence Live and Cochrane Colloquium).

For peer review only

For peer review only - http://bmjopen.bmj.com/site/about/guidelines.xhtml

This study will also include patients or members of the public as key informants, which will allow for better understanding participants' perceived needs and priorities in identifying research gaps to make informed health decisions. Patients/the public will be recruited and identified via patient support groups online, community centres, and public involvement websites such as the peopleinresearch.org platform that involves the public in health research.

We will use purposive sampling to ensure that the perspectives of all identified stakeholder groups are represented. Purposeful sampling is widely used in qualitative research for identifying and selecting information-rich cases, and in this study, further elaboration of the term research gap is needed to better understand the context of the research gaps and methods/practices used to identify and display the research gaps [22, 23].

We anticipate performing about 14 to 28 interviews. This number of interviews will provide for data saturation (i.e., the point when new data do not add to a better understanding of the studied phenomenon but rather repeat what was previously expressed [24]) and also obtain a scope of responses from each stakeholder group. This estimation of interview participants is based on a study involving 60 interviews that showed saturation with 12 interviews, with broader themes apparent after only 6 interviews [25]. The authors noted that factors such as heterogeneity of the sample affect how many interviews are required but concluded that to understand common perceptions and experiences among a group of relatively homogeneous individuals, 12 interviews should suffice[25]. Another study, after examining 25 in-depth interviews, found code saturation after 9 interviews, with the range of thematic issues identified; the authors proposed 16 to 24 interviews to reach saturation (i.e., a richly textured understanding of issues [26]). Therefore, we aim to gather 14 to 28 interviews for our three main categories (health policy, practice and research).

Saturation will be guided by the seven parameters identified by Hennink et al., [26, 27] including the study purpose, population, sampling strategy, data quality, type of codes, code book and saturation goal, and focus retrieved from the study. Each of these parameters will be considered throughout the study.

For peer review only - http://bmjopen.bmj.com/site/about/guidelines.xhtml

Table1. Key informants

Categories	Key informants	Examples	Expected number of interviews
Health policy	Policymakers	Ministry of health officials	2–4
Health practice	Clinicians	Health care professionals (doctors, nurses)	2–4
	Clinical guideline developers	UK National Institute for Health and Care Excellence	2–4
	Public health professionals, Commissioners	National public health bodies	2–4
	Public/patients	Patient forums/groups	2–4
Health research	Researchers	Research institutes/universities<br>Knowledge synthesis research groups<br>Belgian Health Care Knowledge Centre (KCE)<br>Africa Evidence Network<br>Student Forums	2–4
	Funding bodies	UK National Institute for Health Research<br>European Union	2–4

Data collection and recording

Semi-structured interviews will be used for this study. The main reason for selecting semi-structured interviews is to allow for specific areas to be addressed while giving the interviewees the opportunity to reflect on their experiences and perspectives related to defining, identifying, and presenting research gaps that are relevant to them and that may not have been explored or anticipated by the researcher(s) [28].

We will conduct interviews in-person and using teleconference, according to the participant's availability and preference. In-person interviews will be conducted primarily with participants residing or reachable in London, UK, and other participants will be interviewed via teleconference (see Appendix 1 for the interview guide for both the in-person and teleconference interviews). The interviews will be recorded on a digital recorder for face-to-face interviews and electronically for teleconference interviews.

For peer review only - http://bmjopen.bmj.com/site/about/guidelines.xhtml

The guide was developed by focusing on exploring key stakeholder perspectives and experiences with the following key areas:

1) Participant background information
2) Definitions of research gaps
3) Knowledge, perceptions and experiences on methods/practices used to identify and display gaps in health research to inform further health policy, practice and research

These three domains were developed with information from the scoping review to guide the questions. The interview topic guide will be piloted before data collection. It will also be adapted according to key stakeholder groups to ensure that it is meaningful to their background and to gather more relevant information based on their experiences and knowledge [29].

The semi-structured interview guide contains two levels of questions: main themes and follow-up questions. The main themes cover the general content of the research gaps aimed to encourage participants to speak freely about their perceptions, experiences, and practices. Follow-up questions are prompts and probes aimed at following respondents' answers and investigating the raised issues more in-depth. The interview guide covers the main topics of the study, providing a focused structure for the discussion during the interviews. However, it does not need to be strictly followed — the main focus is on providing a setting that encourages respondents to share their perceptions and experiences with research gaps as thoroughly as possible within the constraints of our study aims [30].

All interviews will be transcribed verbatim and anonymised. The lead researcher (LN) will transcribe two interviews to help inform the analytical process, and the other audio files will be transcribed by a professional transcription agency licensed from the University of Liverpool.

Data analysis

We will use analytical categories to describe and explain definitions, experiences and practices reported among the groups of participants. All data relevant to each category (describing research gaps, experience with identifying and displaying research gaps) will be identified and examined to ensure that each data item is checked accordingly.

For peer review only - http://bmjopen.bmj.com/site/about/guidelines.xhtml

Our approach is based on the thematic analysis outlined by Braun and Clarke [31]. The steps include the following: 1) transcription and checking transcripts with recordings for accuracy; 2) open coding from interview responses to be performed by two researchers independently (LN and DH); 3) agreement of initial codes to be discussed among the researchers and an initial codebook developed; 4) the code structure to be used for analysing the remaining responses with openness to including new codes and refining existing ones; and 5) themes and subthemes to be identified from the final code structure and their relationships presented [31].

The initial coding framework for our analysis will start from broad categories identified in the previous scoping review, on which the interviews were structured. Within these broad categories (i.e., describing research gaps, experience with identifying and displaying research gaps), analytic categories will be inductively derived from the data. In this sense, our approach includes both top-down and bottom-up development of analytic categories and themes.

Trustworthiness during thematic data analysis will be ensured by storing raw data systematically, documenting detailed notes about the development and hierarchies of concepts and themes, establishing consensus on themes, providing detailed descriptions of context, and describing the process of coding and analysis [8, 9]. NVivo 12 Pro, a qualitative data analysis software, will be used for data management and analysis.

Ensuring study quality

To further ensure rigour and trustworthiness, this study will be guided by Guba and Lincoln’s concepts for defining and investigating quality in qualitative research that can be considered parallel to quantitative research concepts of validity and reliability [27, 32, 33]. The concepts include credibility, transferability, dependability, confirmability, audit trails and reflectivity. They are interrelated, and thinking through them from the onset and incorporating them in a study will improve the study rigor.

Credibility is defined as the confidence that can be placed in the truth of the research findings [34-36]; it is considered the most important criterion to ensure rigour and trustworthiness. To ensure credibility

For peer review only - http://bmjopen.bmj.com/site/about/guidelines.xhtml

of our study, we will use peer debriefing, which will entail the qualitative lead researcher (LN) seeking support from the senior researcher (DH) to provide scholarly guidance. The feedback will help improve the quality of the inquiry findings [36]. Transferability refers to the extent to which findings of qualitative research can be transferred to other contexts and are useful to people in other settings [21, 36-38]. We aim to address transferability by reporting a rich, detailed description of the key stakeholders' context and location [36, 38]. Dependability is related to whether the research questions are clear and logically connected to the research purpose and design [37]. We aim to achieve dependability by first drafting this protocol to guide our study and future studies with a similar purpose. Confirmability has been related to objectivity or neutrality for establishing that the data and interpretations of the findings are not figments of the inquirer's imagination but are clearly derived from the data, that data collection and interpretations of the study are clearly deliberated from the data and not misinterpreted[37]. We aim to address confirmability by documenting the justification of methodological and analytical choices to illustrate how the data were derived in relation to the study objectives and transparently describing the research steps taken from the start of the project to the development and reporting of the findings. Records of the research path will be kept throughout the study, and de-briefing sessions will be held between the main researcher (LN) and senior researcher (DH). Finally, reflexivity includes examining one's own conceptual lens, explicit and implicit assumptions, preconceptions and values and how these affect research decisions in all phases of qualitative studies. Reflexivity will be achieved by ensuring transparency of the study process by maintaining clear documentation.

Patient or public involvement

There is no patient or public involvement in the design or analysis of this planned study. However, we plan to involve patients/the public in findings that pertain to them and in disseminating study findings. This will be achieved by using patient/public online platforms such as peopleinresearch.org.

DISCUSSION

This study will provide insights into issues related to defining research gaps and methods used to identify and display gaps in health research from perspectives of key stakeholders involved in the

For peer review only - http://bmjopen.bmj.com/site/about/guidelines.xhtml

process. This is a follow-up study of a wider project; the first study was a scoping review exploring methods used to identify and display research gaps reported in scientific publications[1]. The scoping review showed variation and ambiguity in how research gaps are described as well as the methods used to identify and prioritize research gaps. Several of the articles described the development of a framework or tool for identifying and prioritizing research gaps and applying it to a specific topic area as an example for application [1, 2, 7, 39]. There were no evaluations of reproducibility of the method/frameworks identified in the scoping review [1, 7]. Furthermore, despite articles highlighting the existence of research gaps in their studies, very few specifically described the gaps and the causes or the method of identification, so fully understanding the relevance and importance of the research gap to adequately address it is difficult. Our scoping review also primarily found the use of secondary research methods such as systematic reviews and scoping reviews as the most commonly used methods to identify gaps; although other methods were identified, they were inadequately described. The scoping review also showed that besides researchers, different audiences including clinicians, policymakers, funders and patients or the public can benefit from understanding gaps and methods/practices on how to identify and display gaps in health research. This qualitative study aims to go beyond the scientific literature in describing, identifying and displaying gaps in health research and directly talk to people about their understanding and practices. Given the nature of this topic that is not fully explored, there is a need to investigate real practices to be able to develop methodological guidance, taking into consideration the existing literature and on-going practices.

This study has some limitations; one is not including patients/the public in designing the study. Including patients/public perspectives would have benefited the study design by being able to improve the importance and relevance of the findings for this population. One of the main strengths of the study is improving the definition of research gaps and subsequently improving the accurate reporting of research gaps to clearly elucidate the characteristics, which can help in making evidence-based decisions. For example, making a decision based on a research gap contributing to lack of primary research on a specific health problem can differ from a research gap related to lack of secondary research summarizing the research. Hence, all these factors regarding research gaps need to be highlighted if they are known and made explicit when disseminating and communicating research.

Additionally, providing more information on what the gap represents may inform users of evidence on more specific information about the research gap and how it can be addressed more accurately. We anticipate that this study will advance efforts in research and practice on this topic area.

ETHICS and DISSEMINATION

Informed consent will be obtained in accordance with the University of Liverpool Ethics Committee board requirements. Verbal consent will be sought for phone interviews and written consent for in-person interviews. Confidentiality and data protection will be ensured in accordance with the University of Liverpool Ethics Committee board. All participant information will be anonymized, and hard-copy data will be stored in a locked unit. Soft-copy material will be stored in a password-protected file. Upon completion of the study and publication of the study results, all study material will be stored and disposed of according to the rules and regulations of the University of Liverpool. The study protocol will be stored in the data repository Zenodo. The research has obtained ethical approval from the University of Liverpool, UK.

At the end of this research project, the results will be presented at conferences and relevant meetings (e.g., H2020 Project MiRoR). They will also be published in a peer-reviewed journal and as part of a doctoral thesis of the PhD fellow (LN) as well as in professional and lay magazines and presented in workshops at professional events for stakeholder groups and as online materials with good practice examples.

Acknowledgements

We acknowledge the feed-back and support of Daniela Lai and Cristian R. Montenegro in providing feedback on the interview guide. We thank Laura Smales (BioMedEditing, Toronto, ON) for editing the manuscript.

Authors' contributions

LN and DH conceived the study with guidance and feedback from RP and CTM. All authors read and approved the final manuscript.

For peer review only

Funding

This project is a part of a MiRoR (Methods in Research on Research)-funded PhD being undertaken by LN. MiRoR received funding from the European Union's Horizon 2020 research and innovation programme under a Marie Sklodowska-Curie grant (agreement no. 676207).

Conflict of interest

The authors declare no conflicts of interest.

Data Sharing

There are no data in this work.

For peer review only

References

1. Nyanchoka L, Tudur-Smith C, Thu VN, Iversen V, Tricco AC, Porcher R: **A scoping review describes methods used to identify, prioritize and display gaps in health research**. *Journal of Clinical Epidemiology* 2019, **109**:99-110.

2. Robinson KA, Saldanha IJ, McKoy NA: **Development of a framework to identify research gaps from systematic reviews**. *J Clin Epidemiol* 2011, **64**(12):1325-1330.

3. Birte S, Martina V, Ami B, Jennifer S, Marie G: **Evidence & Gap Maps: A tool for promoting evidence informed policy and strategic research agendas**. *Journal of Clinical Epidemiology* 2016, **79**:120.

4. Pham MT, Rajić A, Greig JD, Sargeant JM, Papadopoulos A, McEwen SA: **A scoping review of scoping reviews: advancing the approach and enhancing the consistency**. *Research Synthesis Methods* 2014, **5**(4):371-385.

5. Arksey H, O'Malley L: **Scoping studies: towards a methodological framework**. *International Journal of Social Research Methodology* 2005, **8**(1):19-32.

6. Levac D, Colquhoun H, O'Brien KK: **Scoping studies: advancing the methodology**. *Implementation Science* 2010, **5**(1):69.

7. Carey T, Yon A, Beadles C, Wines R: **Prioritizing future research through examination of research gaps in systematic reviews**. *Prepared for the Patient-Centered Outcomes Research Institute* 2012.

8. Whittemore R, Chao A, Jang M, Minges KE, Park C: **Methods for knowledge synthesis: An overview**. *Heart & Lung: The Journal of Acute and Critical Care* 2014, **43**(5):453-461.

9. Kastner M, Antony J, Soobiah C, Straus SE, Tricco AC: **Conceptual recommendations for selecting the most appropriate knowledge synthesis method to answer research questions related to complex evidence**. *Journal of Clinical Epidemiology* 2016, **73**:43-49.

10. Manson H: **Systematic reviews are not enough: policymakers need a greater variety of synthesized evidence**. *Journal of Clinical Epidemiology* 2016, **73**:11-14.

11. Perrier L, Lightfoot D, Kealey MR, Straus SE, Tricco AC: **Knowledge synthesis research: a bibliometric analysis**. *Journal of Clinical Epidemiology* 2016, **73**:50-57.

12. Pluye P, Hong QN, Bush PL, Vedel I: **Opening-up the definition of systematic literature review: the plurality of worldviews, methodologies and methods for reviews and syntheses**. *Journal of Clinical Epidemiology* 2016, **73**:2-5.

13. Sales A: **Generating and using evidence: reflections from the perspective of implementation**. *Journal of Clinical Epidemiology* 2016, **73**:6-7.

14. Straus SE, Kastner M, Soobiah C, Antony J, Tricco AC: **Introduction: Engaging researchers on developing, using, and improving knowledge synthesis methods: a series of articles describing the results of a scoping review on emerging knowledge synthesis methods**. *Journal of Clinical Epidemiology* 2016, **73**:15-18.

For peer review only

For peer review only - http://bmjopen.bmj.com/site/about/guidelines.xhtml

15. Tricco AC, Antony J, Soobiah C, Kastner M, Cogo E, MacDonald H, D'Souza J, Hui W, Straus SE: **Knowledge synthesis methods for generating or refining theory: a scoping review reveals that little guidance is available**. *Journal of Clinical Epidemiology* 2016, **73**:36-42.

16. Tricco AC, Antony J, Soobiah C, Kastner M, MacDonald H, Cogo E, Lillie E, Tran J, Straus SE: **Knowledge synthesis methods for integrating qualitative and quantitative data: a scoping review reveals poor operationalization of the methodological steps**. *Journal of Clinical Epidemiology* 2016, **73**:29-35.

17. Tricco AC, Soobiah C, Antony J, Cogo E, MacDonald H, Lillie E, Tran J, D'Souza J, Hui W, Perrier L *et al*: **A scoping review identifies multiple emerging knowledge synthesis methods, but few studies operationalize the method**. *Journal of Clinical Epidemiology* 2016, **73**:19-28.

18. Wong G: **Knowledge synthesis approaches–spoilt for choice?** *Journal of Clinical Epidemiology* 2016, **73**:8-10.

19. Chalmers I, Glasziou P: **Avoidable waste in the production and reporting of research evidence**. *The Lancet* 2009, **374**(9683):86-89.

20. Bouma GD, Ling R: *The research process*: Oxford University Press, USA; 2004.

21. Yin RK: **Case study research: Design and methods (applied social research methods)**. *London and Singapore: Sage* 2009.

22. Setia MS: **Methodology Series Module 5: Sampling Strategies**. *Indian journal of dermatology* 2016, **61**(5):505-509.

23. Patton MQ: *Qualitative evaluation and research methods*: SAGE Publications, inc; 1990.

24. Saunders B, Sim J, Kingstone T, Baker S, Waterfield J, Bartlam B, Burroughs H, Jinks C: **Saturation in qualitative research: exploring its conceptualization and operationalization**. *Quality & Quantity* 2017.

25. Guest G, Bunce A, Johnson L: **How Many Interviews Are Enough?: An Experiment with Data Saturation and Variability**. *Field Methods* 2006, **18**(1):59-82.

26. Hennink MM, Kaiser BN, Marconi VC: **Code Saturation Versus Meaning Saturation: How Many Interviews Are Enough?** *Qualitative Health Research* 2016, **27**(4):591-608.

27. Shenton A: *Strategies for Ensuring Trustworthiness in Qualitative Research Projects. Volume 22*; 2004.

28. Britten N: **Qualitative interviews in medical research**. *BMJ* 1995, **311**(6999):251-253.

29. Kvale S, Brinkmann S: *Interviews: Learning the craft of qualitative research interviewing*: Sage; 2009.

30. Gill P, Stewart K, Treasure E, Chadwick B: **Methods of data collection in qualitative research: interviews and focus groups**. *Br Dent J* 2008, **204**(6):291-295.

31. Braun V, Clarke V: **Using thematic analysis in psychology**. *Qualitative Research in Psychology* 2006, **3**(2):77-101.

32. Guba EG, and Yvonna S. Lincoln: *Naturalistic inquiry*; 1985.

For peer review only

For peer review only - http://bmjopen.bmj.com/site/about/guidelines.xhtml

33. Glonti K, Hren D: **Editors' perspectives on the peer-review process in biomedical journals: protocol for a qualitative study**. *BMJ Open* 2018, **8**(10):e020568.

34. Macnee CL, McCabe S: *Understanding nursing research: Using research in evidence-based practice*: Lippincott Williams & Wilkins; 2008.

35. Holloway I, Wheeler S: **Ensuring trustworthiness and quality**. *Holloway I, Wheeler S Research in nursing 2nd Ed Blackwell Publishing, India1996* 2002:250-263.

36. Anney VN: **Ensuring the quality of the findings of qualitative research: Looking at trustworthiness criteria**. *Journal of Emerging Trends in Educational Research and Policy Studies (JETERAPS)*, **5**(2):272-281.

37. Tobin GA, Begley CM: **Methodological rigour within a qualitative framework**. *J Adv Nurs* 2004, **48**(4):388-396.

38. Connelly LM: **Trustworthiness in qualitative research**. *Medsurg Nursing* 2016, **25**(6):435-437.

39. Robinson KA, Saldanha IJ, McKoy NA: **Identification of research gaps from evidence-based guidelines: a pilot study in cystic fibrosis**. *Int J Technol Assess Health Care* 2011, **27**(3):247-252.

For peer review only

Key stakeholders' perspectives and experiences with defining, identifying and displaying gaps in health research: a qualitative study protocol

Appendix 1: Semi-structured interview guide

Date: Interviewer: Archival #:

In person: Teleconference: Start Time: End Time:

Background?

1. Tell me a little about your work, and what you do?
   What does it involve?
2. Experience with using evidence for decision-making in health choices, policymaking, prioritizing research or funding projects?
3. How did you go about making the decision when the evidence was missing, insufficient or inadequate?

Defining research gaps

4. How would you describe the term "research gaps" in your own words?
   Probe based on participant (Researcher, Policy maker, Funder, Health Professional or Public/Patient)
   - *(Research) Can you walk me through how you use evidence to inform future research/research topics?*
   - *(Policy Makers) Can you walk me through how you use research to influence policies?*
   - *(Funders) Can you walk me through how you use research to determine which project to fund?*
   - *(Health Professionals) Can you walk me through how you use research to inform your practice as a health provider?*
   - *(Public/Patients) Can you walk me through how you use research to inform your health decisions?*
     - ✓ What are your thoughts on the importance of identifying research gaps?
     - ✓ What are your thoughts on the causes of research gaps?

Experiences, knowledge and perceived needs with methods used to identify research gaps

5. Could you talk about your views/any experience you have in identifying research gaps?
   - *(Research) For example, if you need to apply for funding, how would you select the study?*
   - *(Policy Makers) For example, if you work in developing policies?*
   - *(Funders) or example, if you need to fund projects, how do you determine which ones to fund?*
   - *(Health professionals) For example, in making decisions between treatment choices in your practice where there is uncertainty?*
   - *(Public/Patients) For example, when making health decisions where there is uncertainty?*

6. Could you tell me more specifically about the methods you used to identify research gaps?
   - ✓ What are some of the strengths of the method(s)/practices you used?
   - ✓ What are some of the challenges you experienced using the method(s) /practices?
7. Looking back on your experience using methods to identify research gaps, what is needed to improve the methods you used to identify research gaps?

Experiences, knowledge and perceived needs with methods used to display/present research gaps

8. Could you describe any experience you have in displaying/presenting research gaps?
9. Could you tell me more about the method(s) you used to display/present research gaps?
   - ✓ What are some of the strengths of the method(s) you used for displaying research gaps?
   - ✓ What are some of the challenges you experienced?
10. Please share any reflection on what you feel is needed to improve the methods you used to display/present research gaps?

General follow-up questions

11. Any additional thoughts you would like to share?

For peer review only - http://bmjopen.bmj.com/site/about/guidelines.xhtml

Key stakeholders' perspectives and experiences with defining, identifying and displaying gaps in health research: a qualitative study protocol

Appendix 2: Participant Information Sheet

Experiences with Methods for Identifying and Displaying Research Gaps

We invite you to take part in our research study. Before you decide whether to participate, you should understand why the research is being done and what it will involve. Please take your time to read the following information carefully and feel free to ask if you need more information or if there is anything that you do not understand. Please also feel free to discuss this with your friends, relatives and anyone else you wish.

What is the purpose of the study?

This study aims to explore the experiences of key stakeholders, including **the public, patients, researchers, clinicians, clinical guideline developers, public health professionals, policymakers and funders**, with methods for identifying and displaying research gaps, to inform **health choices, health practice, future research, policy or funding**. This study aims to help in better understanding the methods used to identify and display research gaps. The overall topic area on methods to identify and display gaps is still not well established, particularly because of no standard definition for the term "research gaps"; therefore a study to better understand the context, as well as the interactions of the factors such as alternative definitions, different audiences and methods used to identify gaps is important to improve our understanding of the audience's needs and the strengths and limitations of methods.

Why have I been chosen to take part?

You have been asked to take part because you are or have been involved in using research, producing research and/or communicating research. Your insight and experience with any methods you have used to identify and display research gaps will be highly appreciated to further guide this topic area.

Do I have to take part?

It is completely up to you whether or not you agree to take part in the study. If you do decide to take part, you will be asked to sign a consent form. If you decide to take part but then change your mind, you are free to do so at any time without giving a reason.

What will happen if I take part?

You will be asked to take part in an interview with a researcher, Linda Nyanchoka, about your experience with and your views of methods for identifying and display research gaps. The interviews will last approximately 20 to 40 minutes or as long as you would like to talk about your experience. With your permission, the interview will be audio-recorded. You can stop the interview at any time, and you do not have to answer a particular question if you don't want to.

Where will the interview take place?

The interview will take place in person at a specific location or over the phone. Participants in the UK have the option of an in-person or teleconference interview, and all other participants will have teleconference interviews at a date and time that is convenient for them.

Are there any risks in taking part?

We do not expect any risks or discomfort associated with this research study. However, if you feel uncomfortable, you can stop the interview at any time, without giving a reason.

For peer review only - http://bmjopen.bmj.com/site/about/guidelines.xhtml

Key stakeholders' perspectives and experiences with defining, identifying and displaying gaps in health research: a qualitative study protocol

Are there any benefits in taking part?

You will be helping develop our understanding of research gaps and methods for identifying and displaying research gaps.

Will my participation be kept confidential?

All the information you give us will be kept strictly confidential. The procedures for handling, processing, storing and destroying the data will comply with the Data Protection Act of 1998.

This means that only the researchers will see what you have said. The audio-recording of your interview will be identified by a code number only. These audio-recordings will be transcribed, and identifying details such as place names and people's names will be removed from the transcripts. We will use quotes from the interviews in the write-up of the study but will ensure that no one can be identified from these quotes.

At the end of the study, the research data, including consent forms, anonymised interview transcripts, field notes and your contact details, will be kept in locked filling cabinets and/or password-protected university computers for up to 10 years.

What will happen to the results of the study?

After the study has finished, the results will be written up as part of the PhD research thesis of Linda Nyanchoka and submitted for examination. The results will also be submitted for publication in an academic journal and presented at conferences.

If you would like to receive a copy of the findings, please let us know by using the contact information provided and we will happily provide you with one.

What will happen if I want to stop taking part?

If you decide at any point that you no longer wish to be part of the study, then you can withdraw without giving a reason. You can also ask for your data to be removed from the study and destroyed.

What if I am unhappy or if there is a problem?

If you are unhappy or if there is a problem, please feel free to let us know by contacting the lead researcher, Linda Nyanchoka, at the University of Liverpool (+33 75 34 29 417; L.Nyanchoka@liverpool.ac.uk). Linda will try to help or put you in touch with someone who can.

If you remain unhappy or have a complaint that you feel you cannot communicate to us, you should contact the Research Governance Officer at the University of Liverpool (0151 794 8290; ethics@liv.ac.uk). When contacting the Research Governance Officer, please provide the name or a description of the study (so that it can be identified), the researcher(s) involved, and the details of the complaint you wish to make.

Who is funding the research?

This research is funded by the European Union's Horizon 2020 research and innovation programme under the Marie Sklodowska-Curie grant (agreement no. 676207). If you want to find out more about the funding body, please contact https://ec.europa.eu/programmes/horizon2020/.

Who is doing this research?

The research and interviews will be conducted by Linda Nyanchoka, a Marie Curie Research Fellow at the University of Liverpool, UK.

For peer review only

Key stakeholders' perspectives and experiences with defining, identifying and displaying gaps in health research: a qualitative study protocol

How can I find out more?

You can get in touch with Linda Nyanchoka, who will be happy to answer any questions you might have:

Department of Biostatistics,

Institute of Translational Medicine

Block F/Waterhouse Buidling,

University of Liverpool,

Liverpool

L69 3BX

Teleconference no.: +33 75 34 29 417

Email address: lnyanchoka@gmail.com or L.Nyanchoka@liverpool.ac.uk

Thank you for taking the time to read this document.

This information sheet is for you to keep

For peer review only

Key stakeholders' perspectives and experiences with defining, identifying and displaying gaps in health research: a qualitative study protocol

Appendix 3: Participant consent form

Researcher: Linda Nyanchoka

Please initial box

1. I confirm that I have read and have understood the information sheet dated **[** **]** for the above study. I have had the opportunity to consider the information, ask questions and have had these answered satisfactorily. ☐

2. I understand that my participation is voluntary and that I am free to withdraw at any time without giving any reason, without my rights being affected. ☐

3. I understand that, under the Data Protection Act 1998, I can at any time ask for access to the information I provide and I can also request the destruction of that information if I wish. ☐

4. I agree for the data I provide to be archived at The University of Liverpool. I understand that other authorised researchers will have access to this data only if they agree to preserve the confidentiality of the information as requested in this form. ☐

5. I agree to take part in the above study. ☐

______________________	________	____________________
Participant name	Date	Signature
______________________	________	____________________
Name of person taking consent	Date	Signature
______________________	________	____________________
Researcher	Date	Signature

For peer review only

Key stakeholders' perspectives and experiences with defining, identifying and displaying gaps in health research: a qualitative study protocol

Principal Investigator

Catrin Tudur-Smith
University of Liverpool
Biostatistics Department
Block F Waterhouse Building
1-5 Brownlow
Liverpool L69 3GL

Tel: +44 (0)151 794 4059
Email: cat1@liverpool.ac.uk

Student Investigator

Linda Nyanchoka
University of Liverpool
Biostatistics Department
Block F Waterhouse Building
1-5 Brownlow Street
Liverpool L69 3GL

Tel: +33 75 34 29 417
Email: L.Nyanchoka@liverpool.ac.uk

The information you have submitted will be published as a report; please indicate whether you would like to receive a copy. ☐

I understand that confidentiality and anonymity will be maintained and it will not be possible to identify me in any publications ☐

I agree for the data collected from me to be used in future research and understand that any such use of identifiable data would be reviewed and approved by a research ethics committee. ☐

I understand and agree that my participation will be audio recorded and I am aware of and consent to your use of these recordings for the following purposes: meeting research aims and goals in exploring methods used to identify and display research gaps. ☐

I understand that the information collected about me will be used to support other research in the future, and may be shared anonymously with other researchers. ☐

I would like my name used and I understand and agree that what I have said or written as part of this study will be used in reports, publications and other research outputs so that anything I have contributed to this project can be recognised. ☐

I understand that my responses will be kept strictly confidential. I give permission for members of the research team to have access to my anonymised responses. I understand that my name will not be linked with the research materials, and I will not be identified or identifiable in the report or reports that result from the research. ☐

For peer review only

For peer review only - http://bmjopen.bmj.com/site/about/guidelines.xhtml

Key stakeholders' perspectives and experiences with defining, identifying and displaying gaps in health research: a qualitative study protocol

I understand and agree that once I submit my data it will become anonymised and I will therefore no longer be able to withdraw my data. ☐

I understand that the fully anonymised data will be held securely at the University of Liverpool and I can request access to the data collected, and/or request that the data is destroyed at any time until the data is submitted for publication. ☐

I understand that other authorised researchers may use my words in publications, reports, webpages, and other research outputs, only if they agree to preserve the confidentiality of the information as requested in this form. ☐

For peer review only

Key stakeholders' perspectives and experiences with defining, identifying and displaying gaps in health research: a qualitative study protocol

Appendix 4: Participant Teleconference Consent Form

Teleconference: Oral Consent Example Script:

Hello, I am Linda Nyanchoka, a PhD student from the University of Liverpool. I will be talking to you about my research project on defining research gaps and on methods to identify and display research gaps in health. Additional information is on the information sheet you have received.

Are you still interested in taking part in the project? *[Await confirmation]*. Now I'd like to confirm some of the details of the project to make sure you are clear about what's involved for you:

- We do not expect any risks or discomfort associated in this research study. However, if you feel uncomfortable, you can stop the interview at any time, without giving a reason.
- You do not have to say yes to take part; you can ask me any questions you want before or during the interview; you can also withdraw at any stage without giving a reason and without any negative consequences.
- You do not have to answer any questions that you do not wish to.
- You are aware that a University of Liverpool Research Ethics committee has approved this research project; for further information email me at L.Nyanchoka@liverpool.ac.uk
- I may use brief quotes of what you say during the interview in the write-up of this study, but they will remain anonymous.
- I will safely store your data electronically in encrypted, secure files. All identifiable data will be destroyed at the end of the study.
- I will audio-record you unless you say that I can't.
- Are you still willing to take part?
  Do you give your permission for me to re-contact you to clarify information?

[Await confirmation] So if you're happy with all of that, and have no more questions, let's start.

Researcher: Linda Nyanchoka

Participant:

Date:

Time:

For peer review only
